# MiR-122 promotes metastasis of hepatoma cells by modulating RBM47-integrin alpha V-TGF-beta signaling

**DOI:** 10.1371/journal.pone.0327915

**Published:** 2025-07-10

**Authors:** Lijun Mao, Hanru Yang, Ning Huang, Yao Li, Ye Sang, Chunxian Zeng

**Affiliations:** 1 Shenzhen Key Laboratory of Viral Oncology, Shenzhen Hospital, Southern Medical University, Shenzhen, China; 2 Shenzhen School of Clinical Medicine, Southern Medical University, Guangzhou, China; 3 Nanfang College, Guangzhou, China; 4 Institute of Precision Medicine, The First Affiliated Hospital, Sun Yat-Sen University, Guangzhou, China; Tabriz University of Medical Sciences, IRAN, ISLAMIC REPUBLIC OF

## Abstract

MiR-122 is a liver-abundant miRNA, which is thought to harbor antitumorigenic activity. Elevated transforming growth factor-β (TGF-β) in hepatocellular carcinoma (HCC) microenvironment is a potent inducer for tumor metastasis. However, the involvement of miR-122 in regulation of TGF-β signaling and its implication in TGF-β-related HCC metastasis remains obscure. In this study, we demonstrated that miR-122 significantly enhanced the activities of the TGF-β pathway reporter, the levels of phosphorylation of Smad2 and Smad3, and the expression of mesenchymal markers (N-cadherin and vimentin) in HCC cells. Notably, miR-122 significantly promoted the migration and invasion *in vitro* and pulmonary metastasis of HCC cells *in vivo*. Mechanism investigations revealed that miR-122 directly suppressed the expression of RBM47, which was a novel RNA binding protein. RBM47 decreased the level of α_v_ integrin (ITGAV) by promoting the degradation of mRNA via interacting with the AU-rich elements in its 3’UTR. Subsequently, the elevated ITGAV induced by miR-122 promoted activation of the latent TGF-β, thereby boosted the TGF-β signaling and then promoted cell motility. Taken together, miR-122 could promote metastasis of hepatoma cells by regulating RBM47-ITGAV-TGF-β signaling. These findings provide new insight into the regulatory network of miR-122, the complexity and robustness of TGF-β pathway and the mechanisms of HCC metastasis.

## Introduction

MicroRNAs (miRNAs) belong a class of small noncoding RNAs that regulate the expression of protein-encoding gene in the post-transcriptional level. They are powerful modulators of various physiological processes including cell proliferation, differentiation, apoptosis and motility [1]. A single miRNA can regulate multiple cellular pathways by targeting a cohort of protein-encoding genes. This property confers miRNAs as promising therapeutic tools for multiple diseases. However, this character of miRNAs is also the main challenge for therapeutic application because their cellular effects are so diverse and need to be interpreted in the various cellular context [2].

MiR-122 is the liver-abundant miRNA, which is frequently downregulated in hepatocellular carcinoma (HCC) tissues [3–5]. miR-122 has been thought to be tumor suppressive miRNA for quite some time, because its deregulation may endue HCC cells with malignant characteristics, including uncontrolled proliferation [4], resistance to apoptosis and chemotherapy [6,7], and the ability of metastasis [8,9]. However, many of the published studies that explored the involvement of miR-122 in hepatocarcinogenesis focused on the tumor cells themselves rather than the context of tumor microenvironment [5,10].

HCC is an inflammation-related cancer and mainly arises from hepatic fibrosis/cirrhosis. A high level of transforming growth factor-β (TGF-β) is usually detected in HCC tissues [11]. The TGF-β cytokine is secreted as a latent complex and is stored in the microenvironment via cross-linking with the extracellular matrix [12]. The major regulation of TGF-β signaling initiation in tissues relies on extracellular activation of the latent TGF-β, in which α_v_ integrin (ITGAV) plays the essential role [12,13]. Once activated, TGF-β binds to the TGF-β receptors and subsequently leads to phosphorylation of Smad2 and Smad3. The activated Smad2/3 cooperates with Smad4 to form complexes, which translocate into nucleus and activate the transcription of the target genes [11]. The TGF-β signaling is frequently activated in HCC and facilitates metastasis by inducing epithelial-mesenchymal transition (EMT) of tumor cells [11]. Although some studies reveal that miR-122 could suppress the mobility of a few HCC cells [5,8,9], little is known about the effect of miR-122 on the TGF-β-related HCC metastasis.

Here, we revealed that miR-122 boosted TGF-β signaling, induced EMT, and significantly promoted migration and invasion of HCC cells *in vitro* and pulmonary metastasis *in vivo*. We further characterized the underlying mechanisms responsible for the pro-metastasis effect of miR-122.

## Materials and methods

The details about reagents and experimental procedures are described in the Supplementary Materials and Methods.

### Cell lines

The HCC cell line SNU-449, the endothelial cell line derived from the metastatic ascites of liver adenocarcinoma SK-Hep-1 and the transformed human embryonic kidney cell line HEK293T were from ATCC. The HCC cell lines (Huh-7 and HLE) and the liver cancer associated fibroblasts were kindly provided by Prof. Shi-Mei Zhuang from Sun Yat-Sen University P.R. China.

### Oligonucleotides and plasmids

We used the following miRNA and small interfering RNA (siRNA) oligonucleotides (Genepharma, Shanghai, China): miR-122 mimics; siTGFBR1 targeting human TGFBR1 (1059−1079 nt, NM_001306210.2) transcript; siITGAV targeting human ITGAV (1136−1156 nt, NM_002210.5) transcript; siRBM47 targeting human RBM47 (1628−1648 nt, NM_001098634.2) transcript. The negative control RNA duplex (NC) for both miR-122 mimics and siRNA was non-homologous to any human genome sequence. The miR-122 inhibitor (anti-miR-122), which is complementary to the sequence of mature miR-122, and its control (anti-NC) consisted of 2’-O-methyl-modified oligonucleotides were purchased from RiboBio (RiboBio, Guangzhou, China). All RNA oligonucleotide sequences are listed in S1 Table.

Lentivirus expression vectors pCDH-miR-122 and pCDH-RBM47 were utilized to express the human miR-122 precursor and RBM47, respectively.

### Cell transfection

A final concentration of 50 nM RNA duplex or 200 nM miRNA inhibitor was transfected using Lipofectamine RNAiMAX (Invitrogen, Carlsbad, CA, USA). Lipofectamine 2000 (Invitrogen) was used to transfect plasmids alone or co-transfect with RNA oligonucleotides.

### Luciferase reporter assay

Luciferase reporter assays were applied to analyze the TGF-β pathway activity, the promoter activities of ITGAV, and verify the miR-122-targeted 3’UTR and the RBM47-targeted region. pRL-TK or pRL-CMV (Promega, Madison, WI, USA), which expresses *Renilla* luciferase, was co-transfected to correct the differences in both transfection and harvest efficiencies. Firefly luciferase activity was normalized to *Renilla* luciferase activity.

### Establishment of HLE subline with stable miR-122 expression

The HLE cell subline with stable miR-122 expression (HLE-miR-122) and its control line (HLE-Ctrl) were established by the lentivirus expression system. The lentiviruses were generated as described previously [14]. HLE cells were infected with lentivirus twice, and qPCR was performed to confirm the stable expression of miR-122 in the HLE-miR-122 subline.

### *In vivo* metastasis assay

The Non-obese C.B-17-scid-IL2rg-/- (NCG) mice which are a highly immunocompromised host model with an NOD (Non-obese diabetic) genetic background were used. Five-weeks old NCG male mice (GemPharmatech, Jiangsu, China) were housed in a temperature- and humidity-controlled vivarium on a 12-hour dark-light cycle with free access to food and water. The hepatic fibrosis was induced as described previously [14]. Briefly, the mice were intraperitoneally injected with CCl_4_ (0.6 ml/kg body weight, mixed with corn oil at 1:4) twice a week for 4 weeks. HLE-Ctrl or HLE-miR-122 cells (1.5x10^6^) were suspended in 150 μl PBS and intravenously injected into the NCG mice with the first 2-weeks CCl_4_ injection (n = 6 for Ctrl group, n = 5 for miR-122 group). No analgesia was needed during injection. The animals were monitored every two days and executed by cervical dislocation 35 days after tumor cell injection. There was no tumor nodule but only micrometastatic foci formed in this model for such short experimental period. Thus, no tumor size was reported. To evaluate the pulmonary metastasis, the lungs were dissected, fixed in formalin, embedded in paraffin and serially sectioned. All experimental procedures involving animals were performed in accordance with the Guide for the Care and Use of Laboratory Animals (NIH publications Nos. 80–23, revised 1996) and the Institutional Ethical Guidelines for Animal Experiments, and were approved by the Ethics Committee of SHSMU on Laboratory Animal Care (No. 2022−101).

### Cross-linked RNA immunoprecipitation assays

RNA immunoprecipitation (RIP) was performed in Huh-7 cells using anti-RBM47 antibody or control IgG isotype. The precipitated RNA was reverse transcribed into cDNA and then subjected to qPCR analysis. The data are presented as fold enrichment of anti-RBM47 antibody relative to the IgG group. The primers used are listed in S1 Table.

### Bioinformatic tool

The starBase (https://rnasysu.com/encori/) and TargetScan (Release 8.0, http://www.targetscan.org/) were used for the prediction of miRNA target genes. The starBase was also used to investigate the expression levels of miR-122 and RBM47 in the HCC samples.

### Statistical analysis

The data were presented as the mean ± standard error of the mean (SEM) from at least three independent experiments. Comparisons between groups were performed using Student’s *t*-test when there were only two groups, or assessed by one-way ANOVA when more than two groups were compared. Two-factor analysis was performed using two-way ANOVA with a post test for subsequent comparisons of individual factors. Statistical analyses were performed with Graphpad Prism 8 (GraphPad Software Inc., San Diego, CA, USA). All statistical tests were two-sided, and *P* < 0.05 was considered to be statistically significant.

## Result

### miR-122 elevates the activity of TGF-β pathway

To assess the activity of TGF-β pathway in four tumor cell lines, a luciferase reporter p-SBE that contains the Smad binding element in the pGL3-basic vector was employed. As shown in (S1A Fig), HLE, SNU-449 and Huh-7 but not SK-hep-1 cells harbored intrinsic TGF-β pathway activity. Moreover, the recombinant TGF-β treatment significantly elevated p-SBE activity in HLE and SNU-449 cells, whereas had no effect in SK-hep-1 cells (S1B Fig), suggesting that TGF-β pathway is active in HLE, SNU-449 and Huh-7 cells, but inactivated in SK-hep-1 cells.

**Fig 1:**
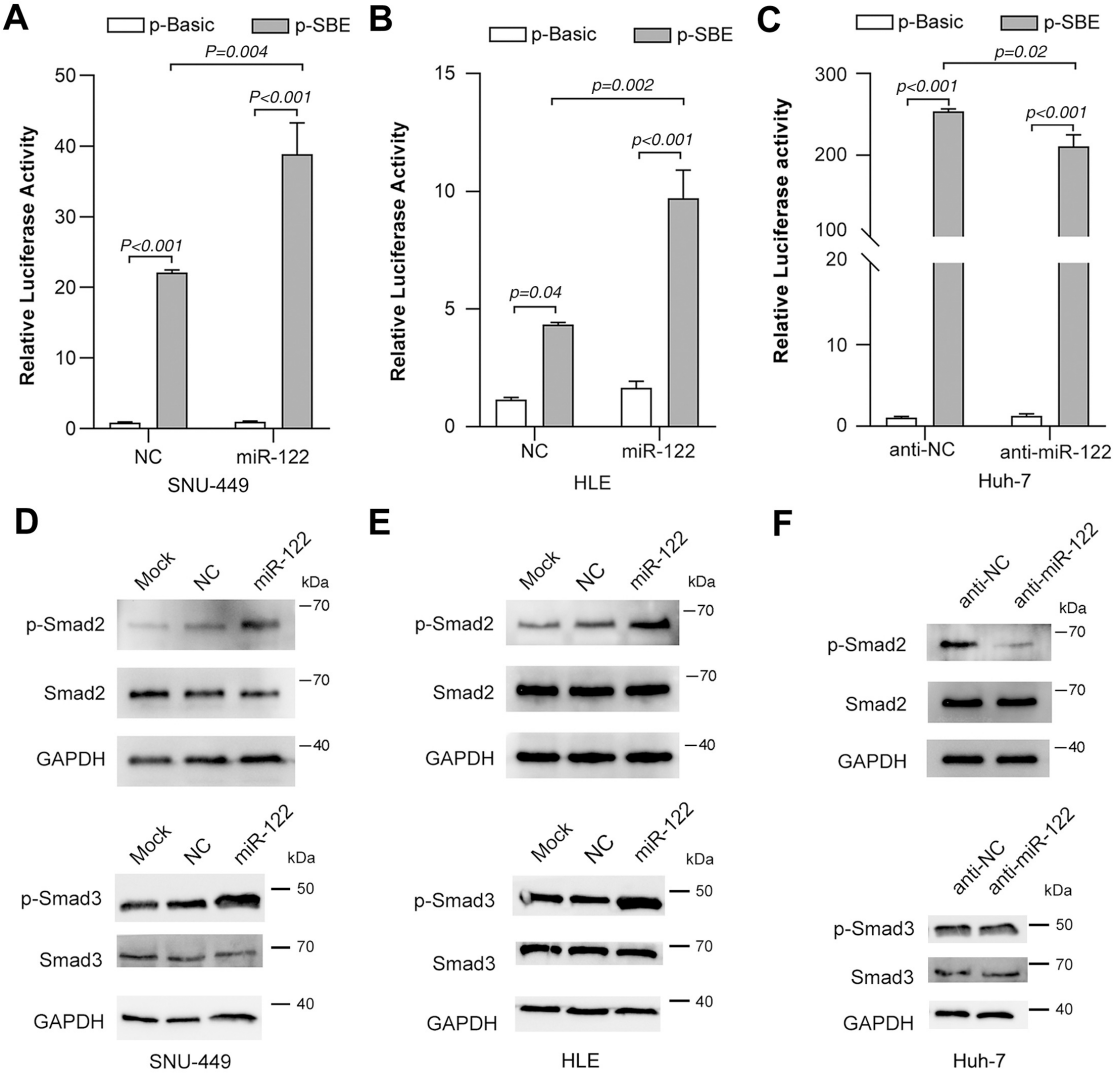
miR-122 elevates the activity of TGF-β pathway. (A, B) Restoration of miR-122 promoted TGF-β pathway activity in SNU-449 (A) and HLE (B) cells. (C) Inhibition of miR-122 reduced TGF-β pathway activity. (D, E) Restoration of miR-122 increased the levels of p-Smad2 and p-Smad3 in SNU-449 (D) and HLE (E) cells. (F) Inhibition of miR-122 reduced the levels of p-Smad2.

To further investigate the effect of miR-122 on TGF-β signaling, we first assessed miR-122 expression in the HCC cell lines with intrinsic TGF-β activity. SNU-449 and HLE cells which displayed lower miR-122 expression were applied in gain-of-function analyses, whereas Huh-7 cells with relatively higher miR-122 level were used in loss-of-function studies (S1C Fig). Interestingly, restoration of miR-122 significantly promoted the p-SBE activities in both SNU-449 (Fig 1A) and HLE cells (Fig 1B). Consistently, inhibiting miR-122 expression with anti-miR-122 (S1D Fig) attenuated p-SBE activity in Huh-7 cells (Fig 1C). Furthermore, we assessed the TGF-β signaling by detecting the phosphorylation of Smad2 (p-Smad2) and Smad3 (p-Smad3), the key markers for TGF-β pathway activation. As expected, introduction of miR-122 significantly elevated both p-Smad2 and p-Smad3 levels in SNU-449 (Fig 1D) and HLE cells (Fig 1E), whereas inhibition of miR-122 dramatically reduced the p-Smad2 levels in Huh-7 cells (Fig 1F). These data implied that miR-122 could promote TGF-β signaling.

### miR-122 promotes EMT and metastasis of HCC cells

We next explored the impact of miR-122 on EMT, one of the initiating steps of tumor cell metastasis elicited by TGF-β signaling. Restoration of miR-122 significantly enhanced the mRNA (S2A–D Fig) and protein levels (S2E, F Fig) of the mesenchymal markers, like the N-cadherin and vimentin in both SNU-449 and HLE cells. The level of epithelial marker E-cadherin in SNU-449 and HLE cells was too low to be detected. On the other hand, inhibition of miR-122 remarkably elevated both the mRNA (S2G Fig) and protein (S2H Fig) levels of E-cadherin in Huh-7 cells. These data implied that miR-122 could promote EMT program.

**Fig 2:**
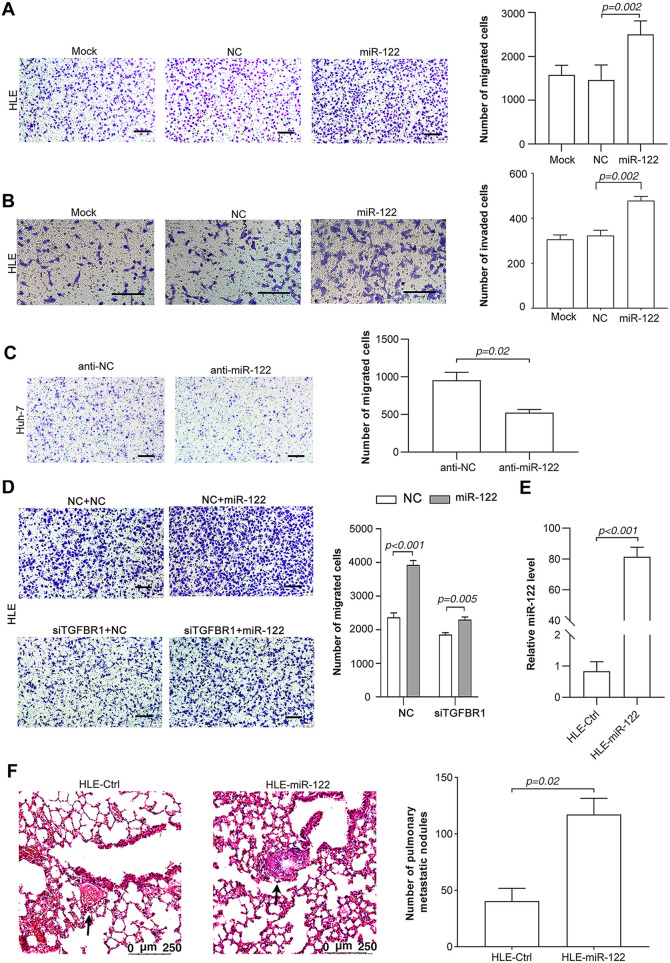
miR-122 promotes HCC cell migration and invasion *in vitro* and metastasis *in vivo.* (A) Restoration of miR-122 promoted migration of HLE cells. (B) Restoration of miR-122 promoted invasion of HLE cells. (C) Inhibition of miR-122 attenuated the migration of Huh-7. (D) Knockdown of TGFBR1 blocked miR-122-promoted cell migration. (E) Detection of miR-122 levels in the HLE stable sublines. (F) The restoration of miR-122 promoted the pulmonary metastases of HCC cells *in vivo*. The arrow indicated the pulmonary metastatic nodule. Scale bar, 250 µm.

We therefore investigated the roles of miR-122 in the metastasis of tumor cells. Consistent with its promoting effect on EMT, restoration of miR-122 significantly promoted the migration (Fig 2A) and invasion (Fig 2B) of HLE and SNU-449 (S3A Fig) cells, without significant effects on the cell viability (S3B Fig). Furthermore, inhibition of miR-122 attenuated the migration of Huh-7 cells (Fig 2C). Intriguingly, ectopic miR-122 obviously inhibited the migration of SK-hep-1 cells (S3C Fig). The opposing roles of miR-122 on migration in different cells might be attributed to the differential TGF-β pathway status in these cell lines (S1A Fig). To further address this hypothesis, we abrogated the TGF-β signaling by neutralizing TGF-β cytokine or silencing TGF-β receptor 1 (TGFBR1) expression. As shown, treatment with anti-TGF-β antibody severely blocked miR-122-induced elevation of p-SBE activity (S3D Fig) and the up-regulation of N-cadherin and vimentin (S3E Fig). Consistently, knockdown of TGFBR1 (S3F Fig) impaired miR-122-promoted cell migration (Fig 2D).

To confirm the pro-metastatic function of miR-122 *in vivo*, a mouse hepatic fibrosis model was employed to produce the endogenous TGF-β. The NCG mice were intraperitoneally injected with CCl_4_ to induced hepatic fibrosis. The HLE subline (HLE-miR-122) which stably expressed miR-122 (Fig 2E) and its control line (HLE-Ctrl) were intravenously injected respectively. Thirty-five days after tumor cell injection, mice were killed and examined. Compared with HLE-Ctrl cells, HLE-miR-122 subline created more metastatic lung nodules (Fig 2F).

Taken together, miR-122 may promote HCC cell EMT and metastasis by elevating TGF-β signaling.

### miR-122 promotes TGF-β signaling by elevating the ITGAV level

To explore the mechanisms responsible for miR-122-promoted TGF-β signaling, exogenous TGF-β cytokine was employed. miR-122 significantly increased p-SBE activity upon treatment with culture supernatant from cancer-associated fibroblast (S4A Fig). Unexpectedly, restoration of miR-122 obviously suppressed the activity of p-SBE induced by recombined TGF-β (S4A Fig). The cell-derived TGF-β is secreted as a latent complex, and needs extracellular activation to function [13] Whereas the recombined TGF-β could trigger signal transduction without ligand activation. Therefore, we proposed that miR-122 could promote TGF-β signaling via enhancing extracellular TGF-β activation.

**Fig 3:**
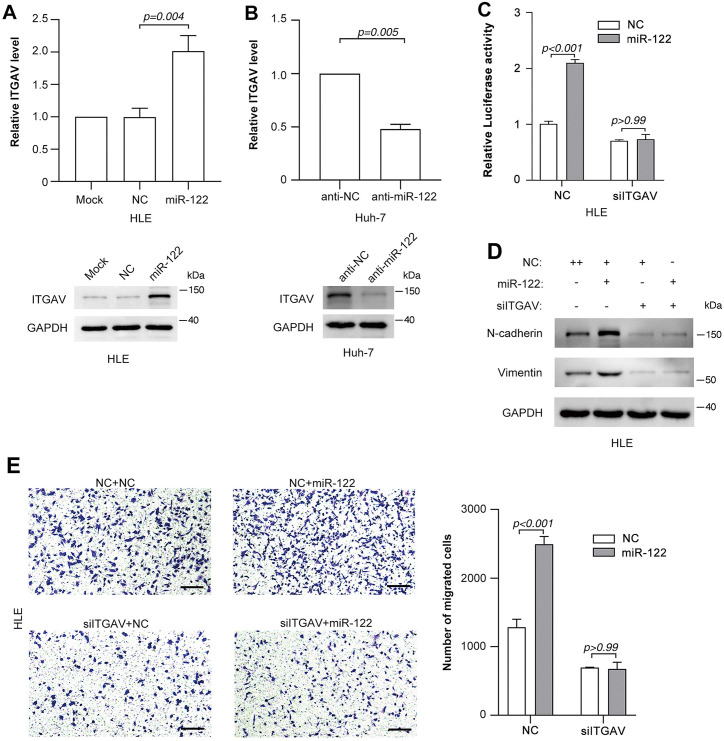
miR-122 promotes TGF-β signaling by elevating ITGAV level. (A) miR-122 elevated ITGAV expression. (B) inhibition of miR-122 suppressed ITGAV expression. (C) Knockdown of ITGAV blocked miR-122-induced elevation of TGF-β pathway activity. (D) siITGAV blocked miR-122-induced elevation of the level of mesenchymal markers. (E) Inhibition of ITGAV abrogated miR-122-promoted cell migration. Scale bar, 250 µm.

It has been reported that multiple integrins, including α_v_β_1_, α_v_β_3_,α_v_β_5_,α_v_β_6_andα_v_β_8_,could activate latent TGF-β [15–18]. We screened the expression levels of integrin subunit α_v_, β_1_, β_3_,β_5_,β_6_andβ_8_, and found that ectopic miR-122 significantly increased the mRNA level of α_v_ subunit (S4B Fig). Moreover, deletion of α_v_ subunit (ITGAV) severely inhibits the activation of TGF-β [13]. Hence, we speculated that miR-122 might modulate ITGAV to promote TGF-β signaling. To address this hypothesis, we further confirmed the effect of miR-122 on the ITGAV expression. As expected, introduction of miR-122 significantly increased both mRNA and protein levels of ITGAV in HLE cells (Fig 3A). Similar results were observed in SNU-449 and Huh-7 cells (S4C, D Fig). Consistently, inhibition of miR-122 repressed the expression of ITGAV in Huh-7 cells (Fig 3B). More importantly, knockdown of ITGAV (S4E Fig) severely blocked the miR-122-induced elevation of p-SBE activity (Figs 3C and S4F), upregulation of the N-cadherin and vimentin levels (Figs 3D and S4G) and the increase of cell migration (Figs 3E and S4H). Taken together, these data suggest that miR-122 promotes TGF-β signaling by elevating the ITGAV level.

### RBM47 is the direct target of miR-122

To investigate the mechanisms underlying the regulation of miR-122 on ITGAV expression, we first constructed the reporter plasmid containing the identified promoter region of ITGAV gene [19]. Interestingly, ectopic miR-122 had no effect on the reporter activity (S5A Fig), implying that miR-122 may increase ITGAV expression at the post-transcriptional level. To test the effect of miR-122 on ITGAV mRNA stability, Huh-7 cells transfected with miR-122 or NC duplex were treated with Dactinomycin, an inhibitor of RNA synthesis. The results showed that overexpression of miR-122 delayed the degradation of ITGAV mRNA (S5B Fig). Furthermore, inhibition of miR-122 led to faster degradation of ITGAV mRNA (S5C Fig). These data indicate that miR-122 may enhance the stability of ITGAV mRNA, therefore increases its expression level. It’s well known that miRNAs suppress the expression of target genes at the post-transcriptional level. And there was no target sequence for miR-122 predicted on the ITGAV mRNA. Hence, it could be inferred that the stability of ITGAV mRNA might be indirectly enhanced by miR-122.

**Fig 4:**
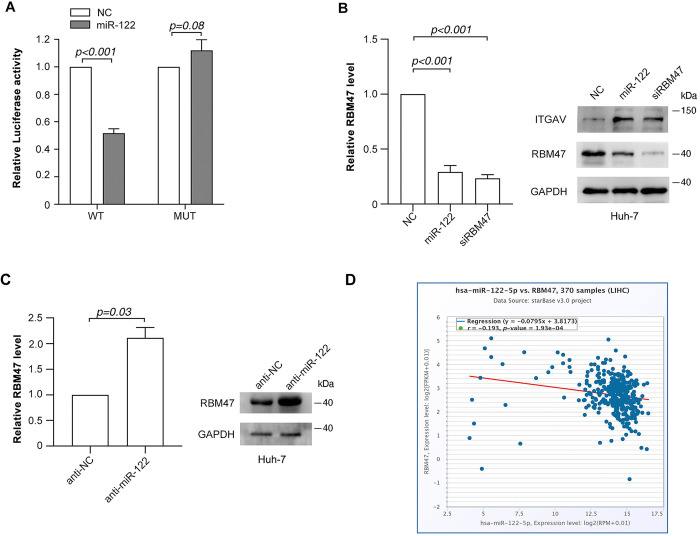
miR-122 targets RBM47 via interacting with its 3’UTR. (A) miR-122 inhibited the activity of luciferase reporter containing the wild-type 3’UTR region of RBM47. (B) Ectopic miR-122 decreased RBM47 expression. (C) Inhibition of miR-122 elevated RBM47 expression. (D) The level of RBM47 was negatively correlated with that of miR-122 in HCC tissues. The gene expression data in the HCC tissues from the starBase were analyzed.

Next, the starBase and TargetScan were used to predict the potential targets of miR-122. RBM47 attracted our interest among the cohort of predicted targets, because RBM47 is a novel RNA binding protein which could modulate target gene expression via cytidine to uridine editing or AU-rich element-mediated RNA decay [20]. We speculated that miR-122 might modulate the stability of ITGAV mRNA by targeting RBM47. As expected, miR-122 suppressed the activity of firefly luciferase with wild-type but not mutant 3’UTR of RBM47 (Figs 4A and S6). Furthermore, both gain-of function (Fig 4B) and loss-of-function (Fig 4C) studies revealed a suppressive effect of miR-122 on the expression of endogenous RBM47. Moreover, the negative correlation between RBM47 and miR-122 expression was determined by the starBase (Fig 4D). These results suggest that miR-122 might inhibit RBM47 expression by directly binding to its 3’UTR.

**Fig 5:**
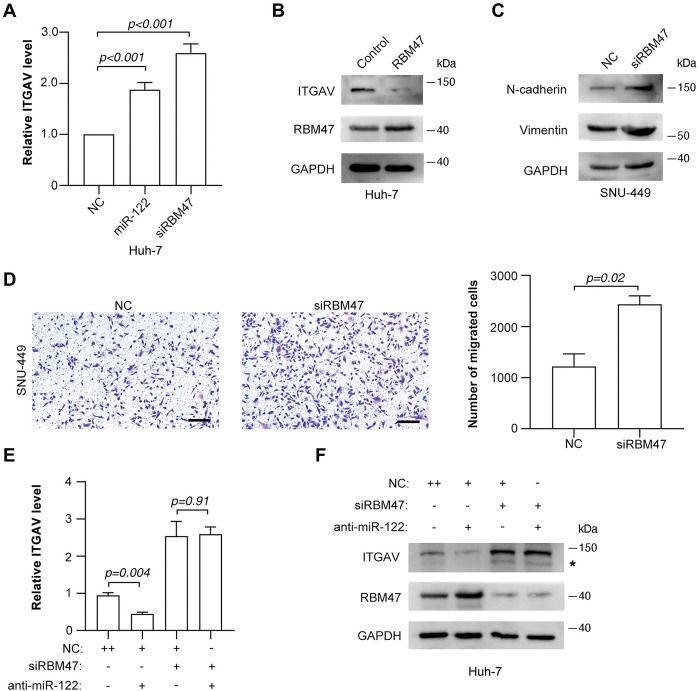
RBM47 inhibits the expression of ITGAV. (A) Inhibition of RBM47 enhanced ITGAV expression. (B) Ectopic RBM47 repressed ITGAV expression. (C) Silencing RBM47 elevated the expression of mesenchymal markers. (D) Knockdown of RBM47 promoted cell migration. Scale bar, 250 µm. (E, F) Knockdown of RBM47 blocked the downregulation of ITGAV induced by miR-122 inhibition. The expression of ITGAV was detected by qPCR assay (E) or immunoblotting (F). * indicates the non-specific bands.

We then evaluated the implication of RBM47 in the regulation of ITGAV expression_._ As expected, silencing RBM47 expression obviously elevated both the mRNA (Fig 5A) and protein (Fig 4B) levels of ITGAV_._ Furthermore, ectopic RBM47 (S7A Fig) significantly repressed ITGAV expression (Fig 5B). Moreover, silencing RBM47 mimicked the effect of miR-122 on the expression of N-cadherin and vimentin (Figs 5C and S7B) and the cell migration (Figs 5D and S7C). More importantly, knockdown of RBM47 severely blocked the downregulation of ITGAV induced by miR-122 inhibition (Fig 5E, F). These data indicate that RBM47 might be the functional target of miR-122 which mediates its regulation of ITGAV expression and TGF-β signaling.

### RBM47 modulates ITGAV mRNA stability via AU-rich elements

**Fig 6:**
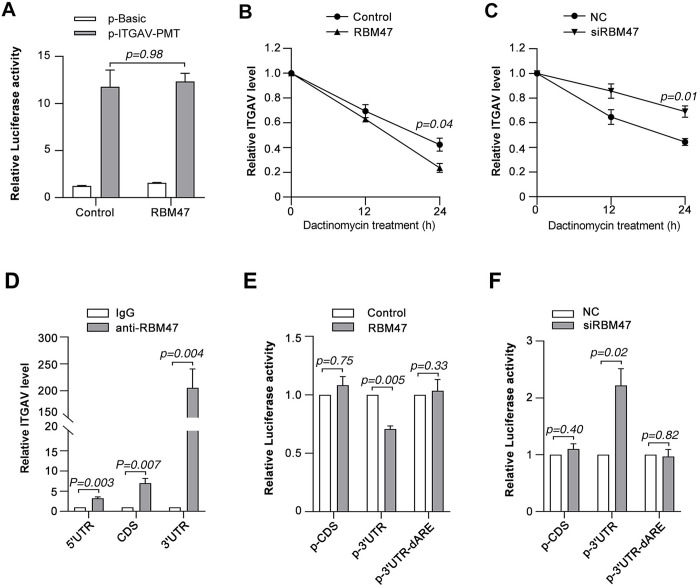
RBM47 modulates ITGAV mRNA stability *via* interacting with its 3’UTR. (A) RBM47 had no effect on the ITGAV promoter activity. (B) Ectopic RBM47 promoted the decay of ITGAV mRNA. (C) Knockdown of RBM47 delayed the degradation of ITGAV mRNA. (D) RBM47 bound to the ITGAV mRNA. (E) RBM47 inhibited the activity of luciferase reporter containing the wild-type 3’UTR region of ITGAV. (F) siRBM47 elevated the activity of luciferase reporter containing the wild-type 3’UTR region of ITGAV.

To investigate the mechanism underlying the regulation of ITGAV level by RBM47, we first tested whether RBM47 affected its transcription. Ectopic RBM47 had no effect on the ITGAV promoter reporter activity (Fig 6A), suggesting that RBM47 does not affect the transcription of ITGAV. Furthermore, ectopic RBM47 promoted the decay of ITGAV mRNA (Fig 6B). Consistently, knockdown of RBM47 significantly delayed the degradation of ITGAV mRNA (Fig 6C), suggesting that RBM47 regulates the half-life of ITGAV mRNA.

AU-rich elements (AREs) are classical motifs which RNA binding proteins can bind to and subsequently regulate the stability of target mRNA. Interestingly, multiple AREs were predicted among the 3’UTR of ITGAV mRNA (S8 Fig). Hence we speculated that RBM47 might promote the ARE-mediated degradation of ITGAV mRNA. To test this hypothesis, we first performed RIP analysis. Anti-RBM47 antibody pulled down a greater amount of ITGAV mRNA than the control IgG, especially at the 3’UTR region (Fig 6D), implying that RBM47 can directly interact with the 3’UTR of ITGAV mRNA. Furthermore, The 3’UTR sequence rich in AREs and the coding sequence of ITGAV mRNA were cloned in the 3’UTR region of luciferase, respectively. Consistent with the change of endogenous mRNA level, ectopic RBM47 suppressed the activity of reporter containing the 3’UTR sequence (p-3’UTR) (Fig 6E), and knockdown of RBM47 elevated the activity of p-3’UTR (Fig 6F). However, manipulation of RBM47 had no effect on the reporter containing the coding sequence (p-CDS) or the mutant 3’UTR (p-3’UTR-dARE) without the predicted AREs (Fig 6E, F). These data demonstrate that RBM47 could promote the degradation of ITGAV mRNA via ARE in its 3’UTR region.

## Discussion

Most of the previous publications about the implication of miR-122 in hepatocarcinogenesis reveal the anticarcinogenic effects of miR-122 [5]. In this study, we provided new insights into the regulatory network of miR-122, and revealed its unexpected role in promoting the activation of TGF-β signaling and the metastasis of HCC.

It has been reported that miR-122 could suppress HCC cell invasion by inhibiting the expression of ADAM17 [5] and RhoA [8], and impairing Wnt pathway-activated EMT [9]. In this study, we demonstrated that miR-122 elevated TGF-β signaling, and subsequently promoted HCC cell EMT and metastasis. The opposite effects of miR-122 on HCC metastasis between our study and previous reports might be attributed to the discrepancy of cell models used in the researches. Many of the previous studies employed Sk-hep-1 cell line as model [5,8], which has marginal TGF-β signaling activity, even upon TGF-β stimulation. We employed SNU-449, HLE, Huh-7 and SK-hep-1 cells in this study. We also showed that miR-122 repressed the migration of SK-hep-1 cells, which was consistent with the previous reports [5,8]. However, in SNU-449, HLE and Huh-7 cells which harbored activated TGF-β pathway, miR-122 could promote TGF-β induced-EMT and cell migration. Hence, the effects of miR-122 on cell migration might depend on the status of TGF-β pathway in the HCC cells. The vital property of miRNA is that a single miRNA can direct multiple cellular pathways. According to our results, compared to ADAM17, Rho family and Wnt pathway, TGF-β signaling might be the predominant pathway to modulate HCC cell migration. One of the prominent features of HCC is the heterogeneity, which is closely related to the diverse molecule pathways disordered in HCC patients. Our results imply that, the potential treatment strategies using miR-122 and the therapeutic effects might depend on the molecular classification of the individuals, especially the status of TGF-β pathway.

Recent study has demonstrated that miR-122 targets TGF-β1 5’UTR and inhibits its expression in human hepatoma cells [21]. Moreover, miR-122 could suppress the TGF-β pathway by directly inhibiting TGFBR2 in the skeletal muscle cells [22]. Our study disclosed that miR-122 might enhance TGF-β signaling in HCC cells by elevating the expression of ITGAV, which is the core molecular participated in the extracellular activation of the latent TGF-β complex [13]. Collectively, miR-122 could regulate multiple aspects of TGF-β signal transduction, including inhibiting the expression of TGF-β1 and the receptor, and promoting the activation of latent TGF-β. We are also aware of that miR-122 could suppress TGF-β-induced expression of alpha smooth muscle actin and α1 type I collagen in both hepatic stellate cells and fibroblasts, and thereby inhibits hepatic fibrogenesis [14]. In this case, miR-122 directly inhibits the expression of serum response factor, a downstream transcription factor of TGF-β signaling which mediates the transactivation of the fibrosis-related genes [14].

RBM47 is a novel multifunctional RNA-binding protein which could regulate several aspects of RNA biogenesis including editing, splicing and stability [20]. RBM47 has been reported to preferentially bind to the 3’UTR of its target mRNAs, and subsequently stabilize the mRNAs [23–25]. We found that RBM47 could interact with the 3’UTR of ITGAV. Contrarily, we demonstrated that RBM47 promoted the decay of ITGAV mRNA via the AREs. It’s well known that a cohort of RNA-binding proteins such as TTP and HuR could bind to AREs at the 3’UTR of mRNAs and control their fate [26]. Although whether RBM47 has intrinsic capability to degrade RNA molecule is still unknown, it’s probably that RBM47 might promote the decay of ITGAV mRNA by recruiting or/and stabilizing RNA-binding proteins to form RNA decay machinery. Our results exhibit a novel function of RBM47 and provide new insight into the regulation of ITGAV expression at post-transcriptional level. However, the detailed mechanisms merit further investigation.

**Fig 7:**
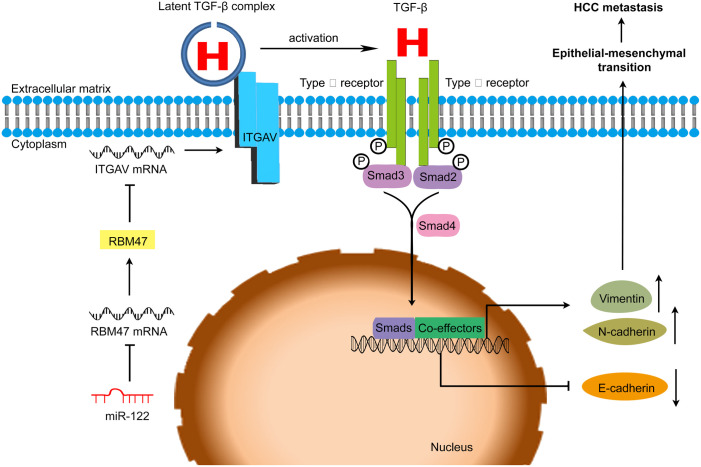
Schematic overview of a novel miR-122-RBM47-ITGAV-TGF-β signaling.

Taken together, we identified a novel miR-122-RBM47-ITGAV-TGF-β regulatory axis: miR-122 suppresses RBM47 expression, subsequently elevates ITGAV level and then promotes TGF-β signaling. At the normal stage or the early phase of HCC, TGF-β signaling mainly suppresses the proliferation of hepatocytes. This regulatory axis contributes to maintaining the homeostasis of hepatocytes. Downregulation of miR-122 at these stages could endow HCC cells with growth advantage. However, the major effect of TGF-β signaling switches from cell growth inhibition to enhancing cell motility during HCC development. Reintroduction of miR-122 might elevate TGF-β signaling, and subsequently promote HCC cell EMT and metastasis (Fig 7). These findings substantially expand our understanding about the regulatory network of miR-122, and provide new insight into the complexity and robustness of TGF-β pathway and the molecular mechanisms of metastasis.

## Supporting information

S1 Fig Detection of TGF-β pathway activities and miR-122 levels in HCC cells. (A) Detection of TGF-β pathway activity using dual luciferase assay. (B) Detection of TGF-β pathway activities in HCC cell lines treated with TGF-β1. (C) The miR-122 levels in HCC cell lines. (D) Inhibition of endogenous miR-122 level.
(TIF)

S2 Fig miR-122 promotes EMT of HCC cells. (A-F) Restoration of miR-122 promotes the expression of mesenchymal markers. The expression levels of N-cadherin and vimentin in the SNU-449 (A, C, E) or HLE (B, D, F) cells were detected by qPCR assay (A-D) or immunoblotting (E, F). (G, H) Inhibition of miR-122 elevated the expression of E-cadherin. The level of E-cadherin in Huh-7 cells was measured by qPCR assay (G) or immunoblotting (H).
(TIF)

S3 Fig Inhibition of TGF-β signaling blocks miR-122-induced EMT and cell migration. (A) Restoration of miR-122 promoted migration of SNU-449 cells. (B) Introduction of miR-122 did not affect cell viability. (C) Restoration of miR-122 inhibited migration of Sk-hep-1 cells. (D) Neutralizing TGF-β blocked miR-122-induced elevation of p-SBE activity. (E) TGF-β neutralizing antibody blocked miR-122-induced up-regulation of N-cadherin and vimentin. * indicates the non-specific bands. (F) Knockdown of TGFBR1. Scale bar, 250 µm.
(TIF)

S4 Fig ITGAV mediated the elevation of TGF-β signaling induced by miR-122. (A) The effect of miR-122 on TGF-β pathway activity induced by TGF-β from different origins. (B) The effects of miR-122 on the expression level of integrin subunits. (C, D) miR-122 elevated ITGAV expression in SNU-449 (C) and Huh-7 (D) cells. (E) Knockdown of ITGAV. (F) Knockdown of ITGAV blocked miR-122-induced elevation of TGF-β pathway activity. (G) Inhibition of ITGAV repressed miR-122-induced elevation of the mesenchymal markers level. (H) Knockdown of ITGAV abrogated miR-122-promoted cell migration. Scale bar, 250 µm.
(TIF)

S5 Fig miR-122 enhances the stability of ITGAV mRNA. (A) miR-122 had no effect on the ITGAV promoter activity. (B) Restoration of miR-122 delayed ITGAV mRNA decay. (C) Inhibition of miR-122 promoted the degradation of ITGAV mRNA.
(TIF)

S6 Fig miR-122 and its putative binding sequences in the 3’UTR of *RBM47.* The wild-type and mutant 3’UTR segment of *RBM47* and miR-122 sequence were shown. Mutations were generated in the complementary site that binds to the seed region of miR-122.
(TIF)

S7 Fig RBM47 modulates EMT and cell migration. (A) Overexpression of RBM47. (B) Silencing RBM47 promoted the levels of the mesenchymal markers. (C) siRBM47 promoted HLE cell migration. Scale bar, 250 µm.
(TIF)

S8 Fig The predicted AREs in the 3’UTR of ITGAV mRNA. (TIF)

S1 Table Sequences of RNA and DNA Oligonucleotides. (DOCX)

S1 File Raw data for graphs. (ZIP)

S2 File S1_raw_images. (PPTX)

S3 File Supplementary materials and methods. (DOC)
