# Supplementary figures and images for: MiR-122 promotes metastasis of hepatoma cells by modulating RBM47-integrin alpha V-TGF-beta signaling
Source: PLoS One. 2025 Jul 10;20(7):e0327915. doi: 10.1371/journal.pone.0327915 (PMC12244532; doi:10.1371/journal.pone.0327915)

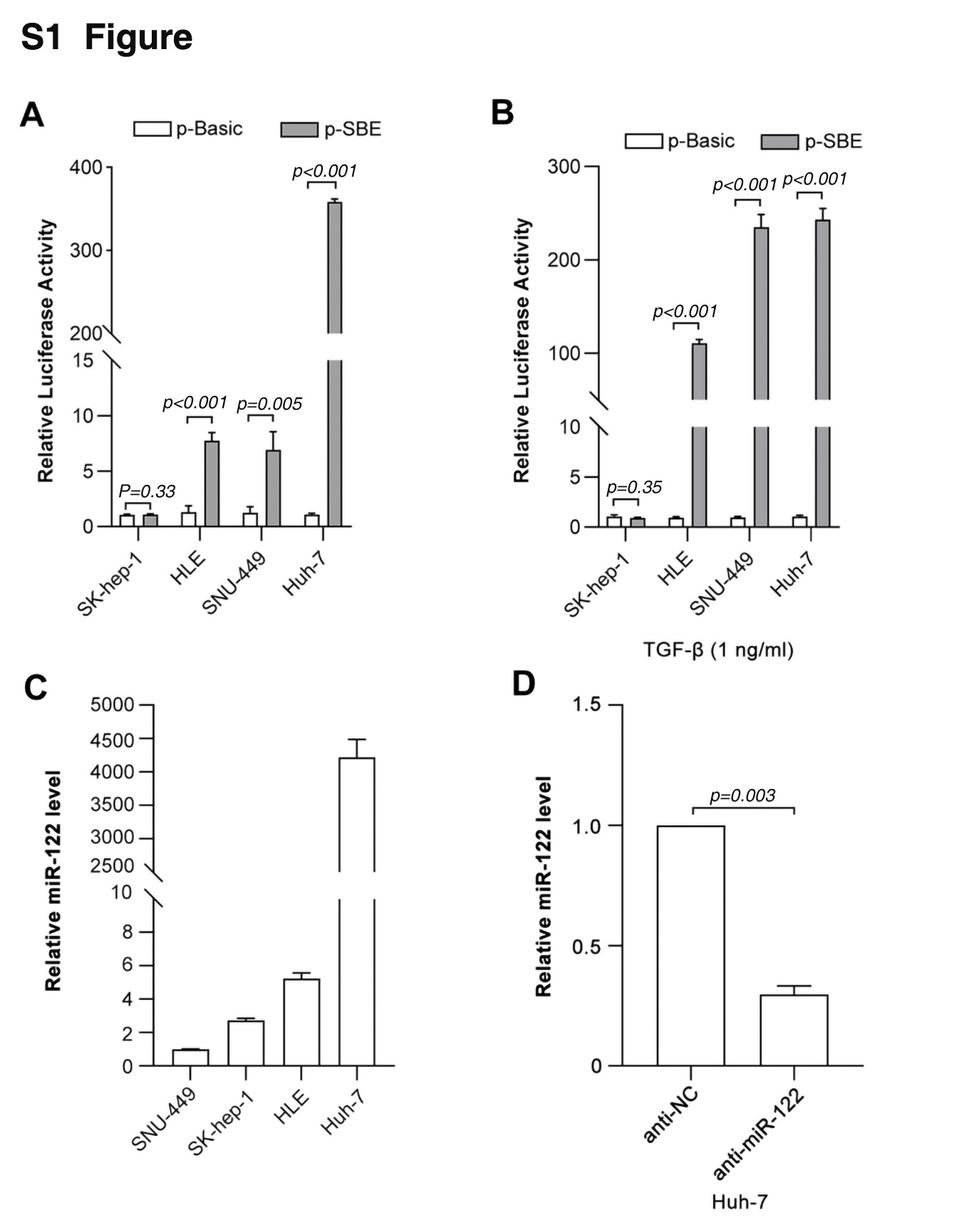

Supplement: S1 Fig — (A) Detection of TGF-β pathway activity using dual luciferase assay. (B) Detection of TGF-β pathway activities in HCC cell lines treated with TGF-β1. (C) The miR-122 levels in HCC cell lines. (D) Inhibition of endogenous miR-122 level. (TIF) [file pone.0327915.s001.tif]

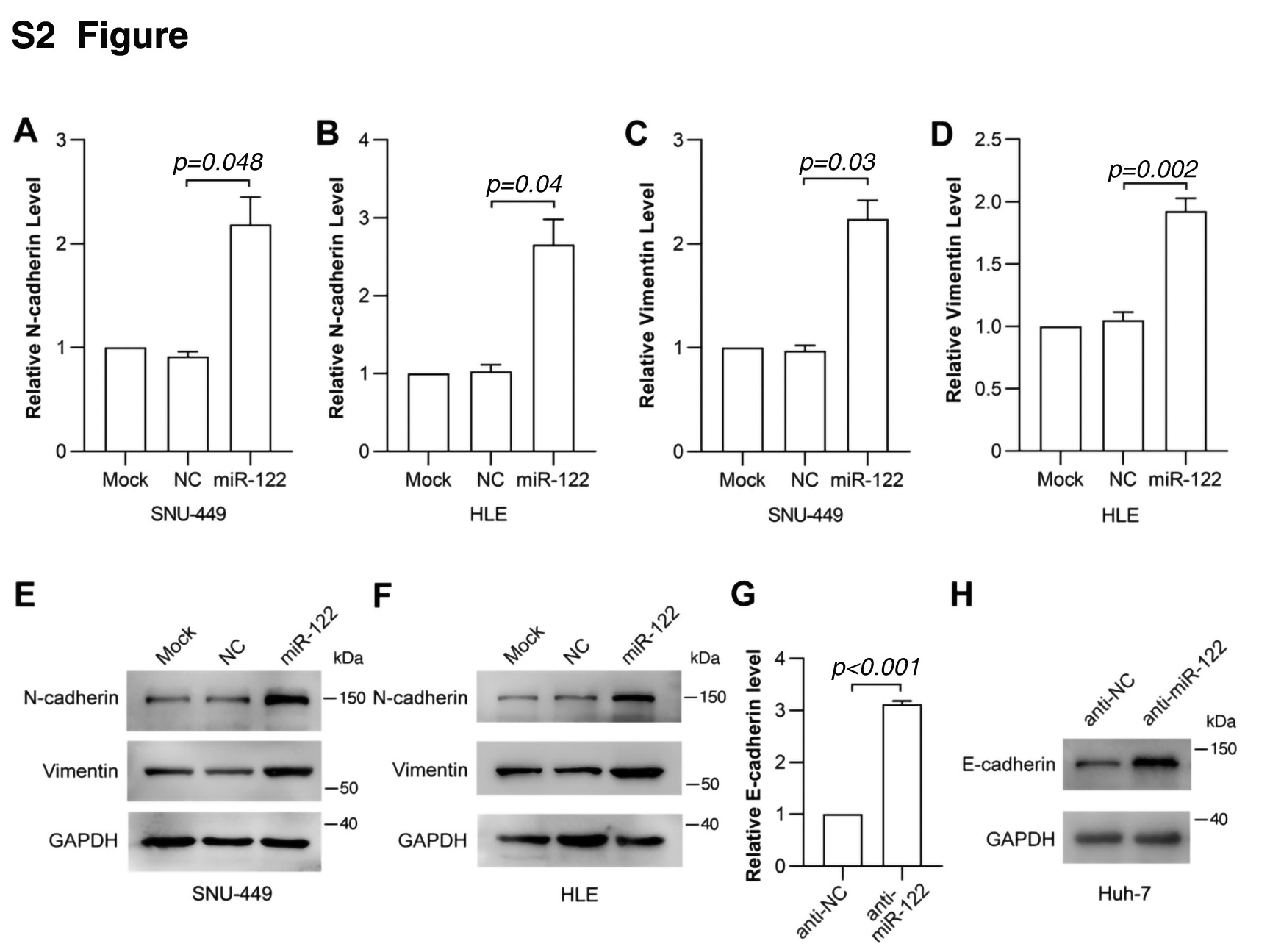

Supplement: S2 Fig — (A-F) Restoration of miR-122 promotes the expression of mesenchymal markers. The expression levels of N-cadherin and vimentin in the SNU-449 (A, C, E) or HLE (B, D, F) cells were detected by qPCR assay (A-D) or immunoblotting (E, F). (G, H) Inhibition of miR-122 elevated the expression of E-cadherin. The level of E-cadherin in Huh-7 cells was measured by qPCR assay (G) or immunoblotting (H). (TIF) [file pone.0327915.s002.tif]

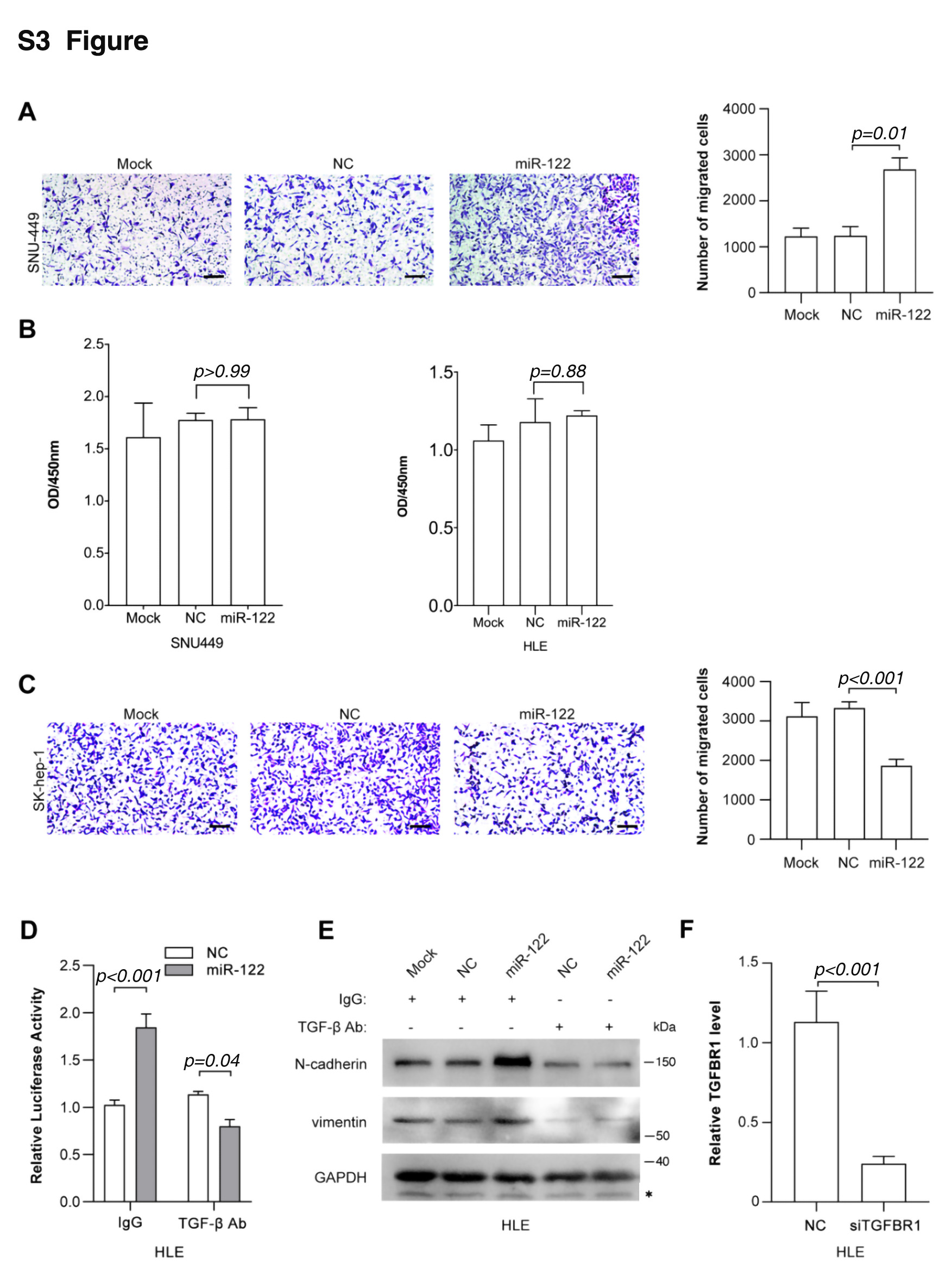

Supplement: S3 Fig — (A) Restoration of miR-122 promoted migration of SNU-449 cells. (B) Introduction of miR-122 did not affect cell viability. (C) Restoration of miR-122 inhibited migration of Sk-hep-1 cells. (D) Neutralizing TGF-β blocked miR-122-induced elevation of p-SBE activity. (E) TGF-β neutralizing antibody blocked miR-122-induced up-regulation of N-cadherin and vimentin. * indicates the non-specific bands. (F) Knockdown of TGFBR1. Scale bar, 250 µm. (TIF) [file pone.0327915.s003.tif]

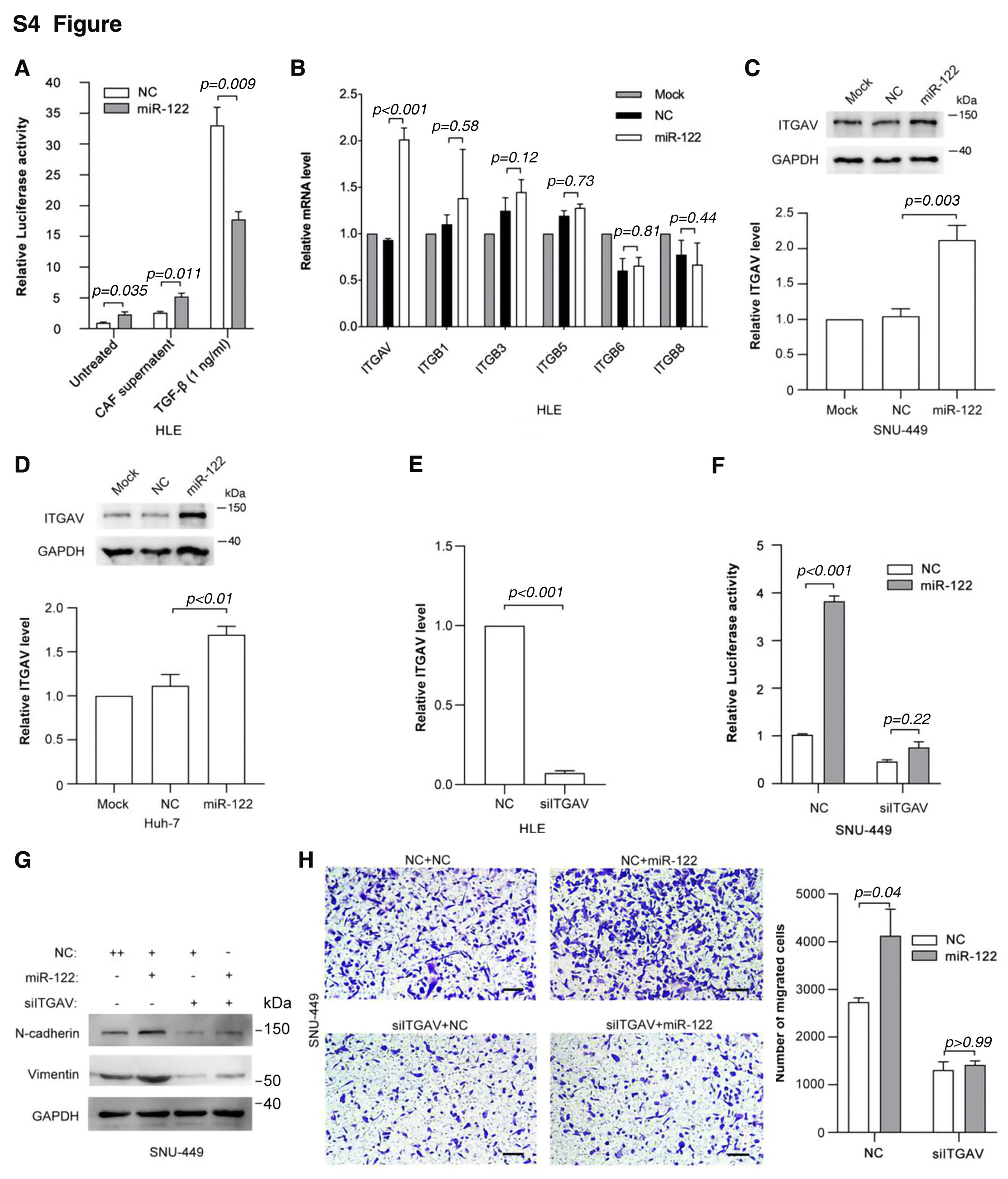

Supplement: S4 Fig — (A) The effect of miR-122 on TGF-β pathway activity induced by TGF-β from different origins. (B) The effects of miR-122 on the expression level of integrin subunits. (C, D) miR-122 elevated ITGAV expression in SNU-449 (C) and Huh-7 (D) cells. (E) Knockdown of ITGAV. (F) Knockdown of ITGAV blocked miR-122-induced elevation of TGF-β pathway activity. (G) Inhibition of ITGAV repressed miR-122-induced elevation of the mesenchymal markers level. (H) Knockdown of ITGAV abrogated miR-122-promoted cell migration. Scale bar, 250 µm. (TIF) [file pone.0327915.s004.tif]

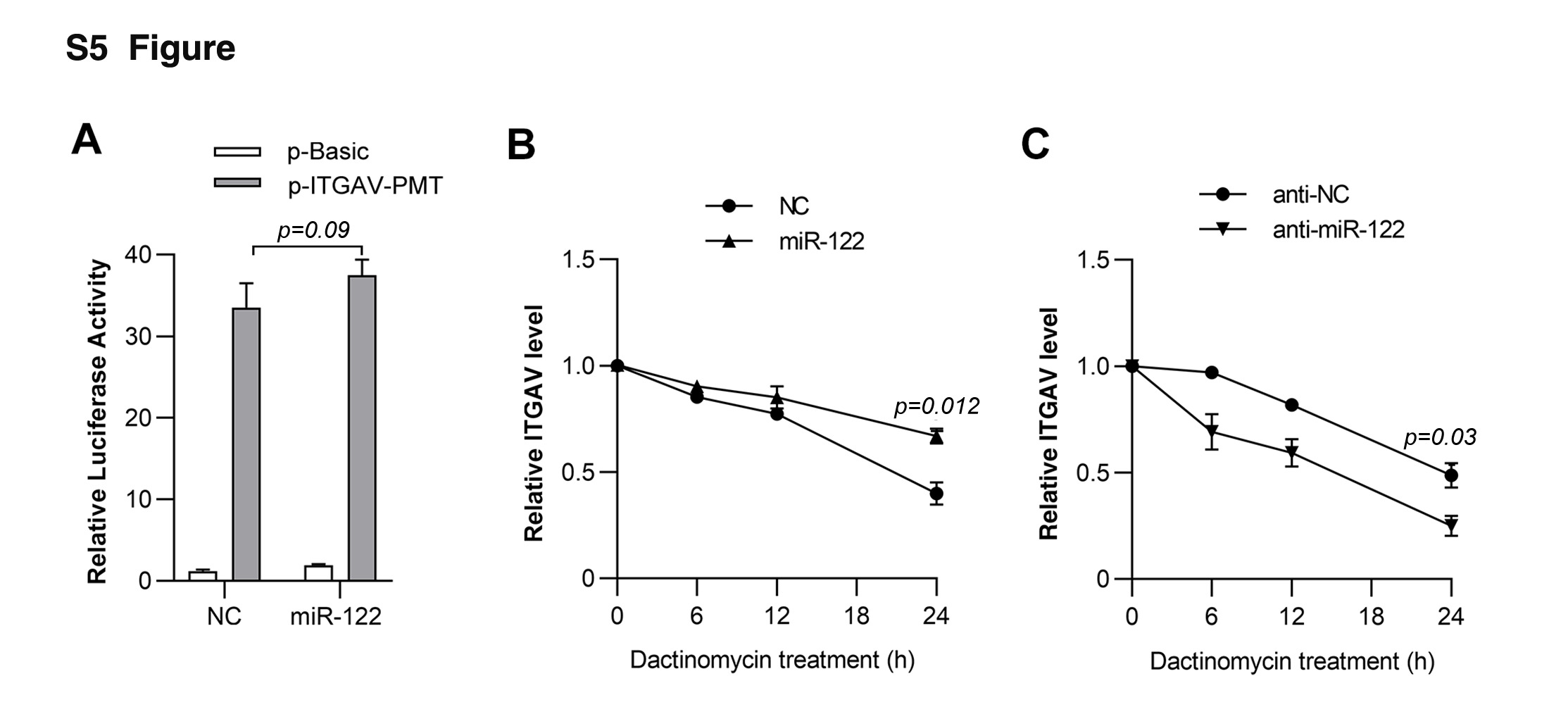

Supplement: S5 Fig — (A) miR-122 had no effect on the ITGAV promoter activity. (B) Restoration of miR-122 delayed ITGAV mRNA decay. (C) Inhibition of miR-122 promoted the degradation of ITGAV mRNA. (TIF) [file pone.0327915.s005.tif]

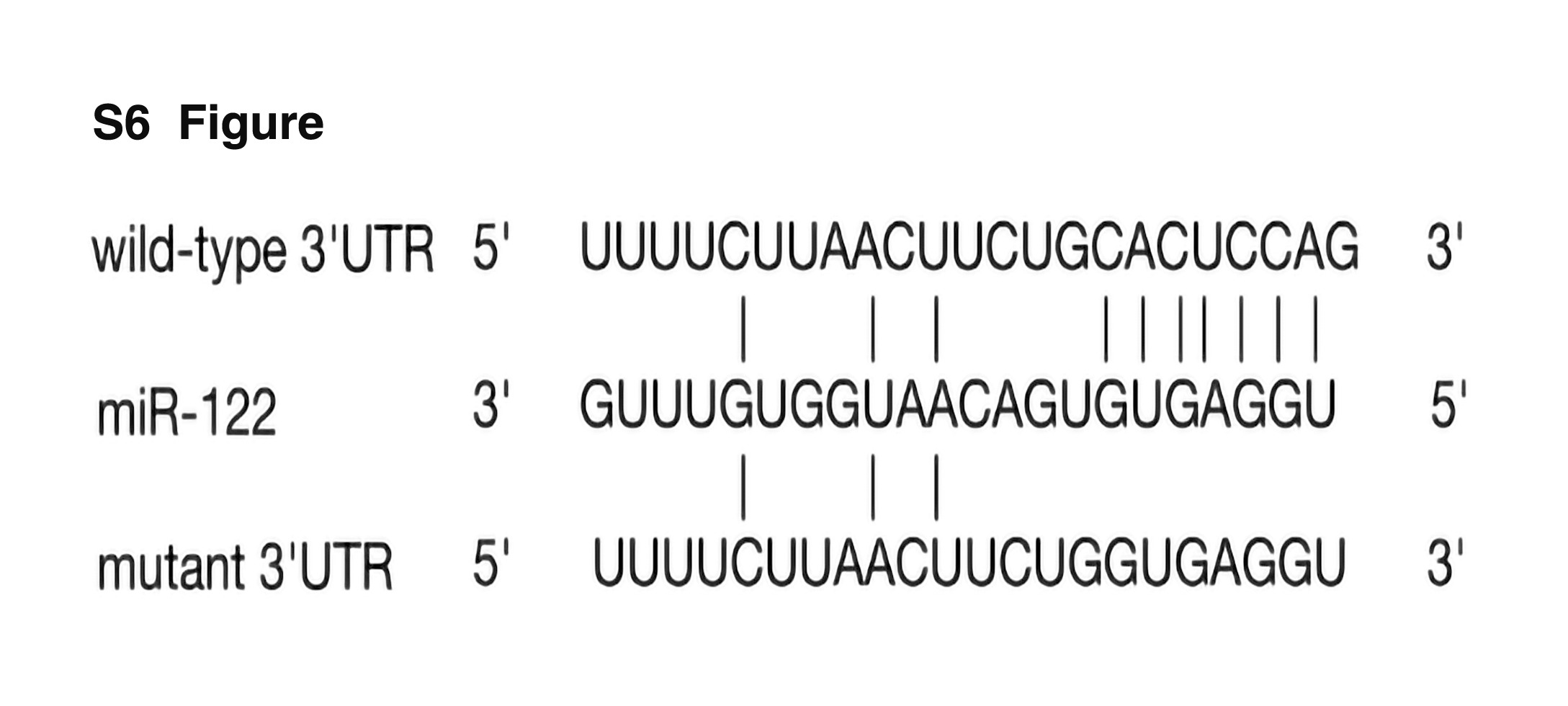

Supplement: S6 Fig — The wild-type and mutant 3’UTR segment of RBM47 and miR-122 sequence were shown. Mutations were generated in the complementary site that binds to the seed region of miR-122. (TIF) [file pone.0327915.s006.tif]

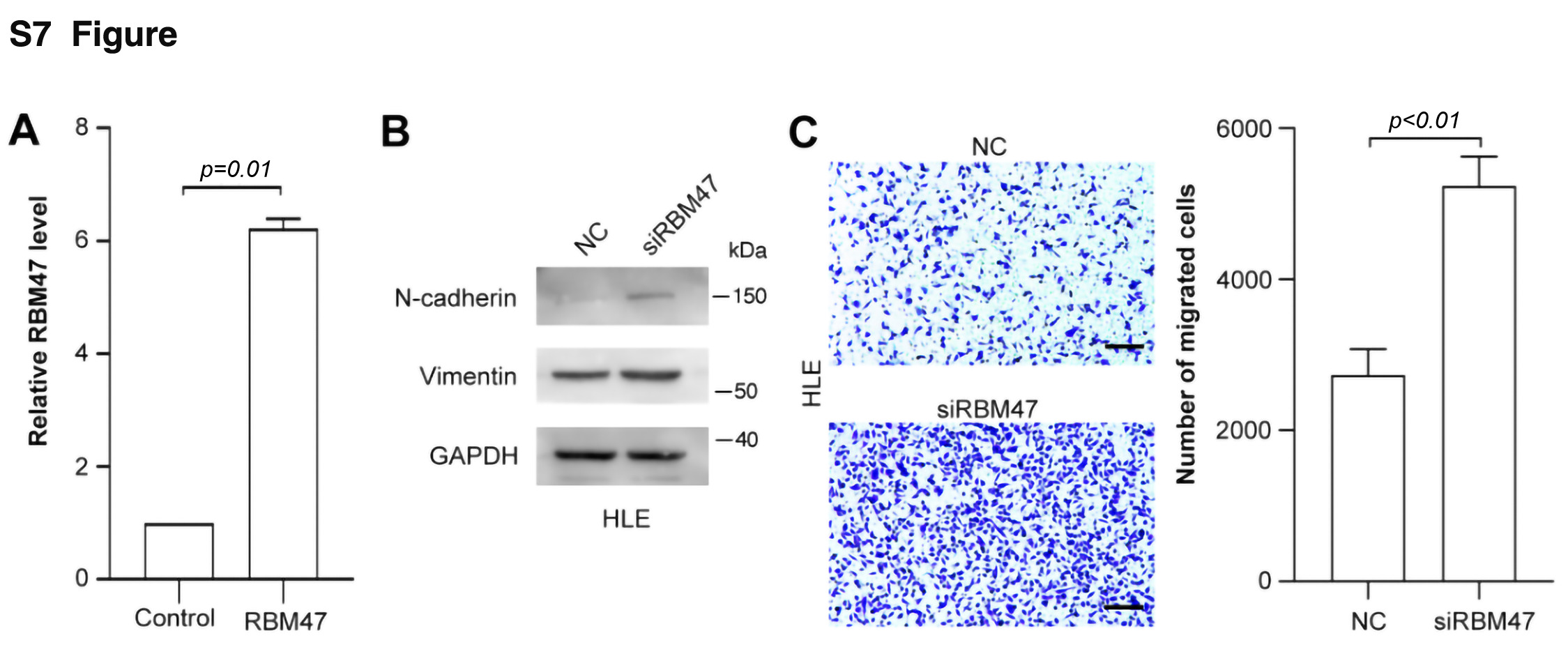

Supplement: S7 Fig — (A) Overexpression of RBM47. (B) Silencing RBM47 promoted the levels of the mesenchymal markers. (C) siRBM47 promoted HLE cell migration. Scale bar, 250 µm. (TIF) [file pone.0327915.s007.tif]

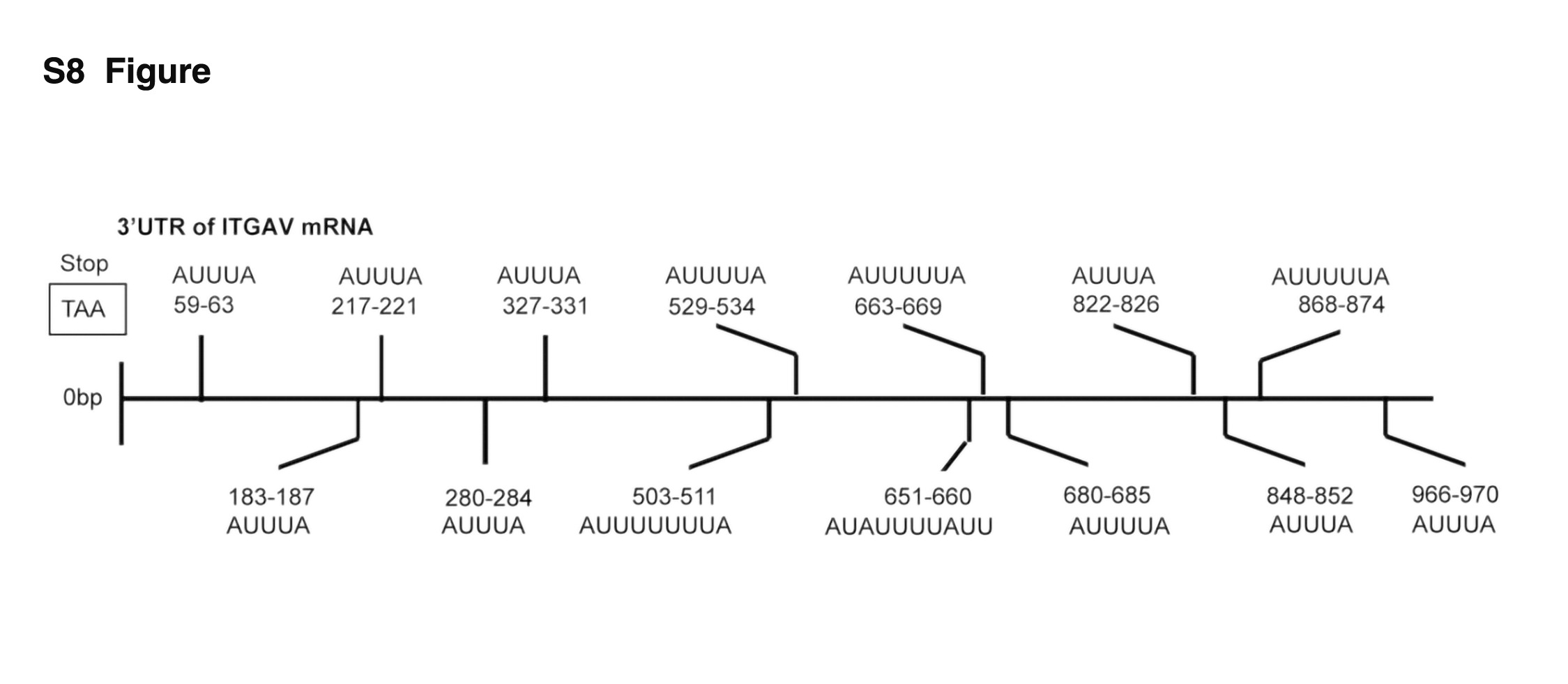

Supplement: S8 Fig — (TIF) [file pone.0327915.s008.tif]
