# Supplementary material for: MiR-122 promotes metastasis of hepatoma cells by modulating RBM47-integrin alpha V-TGF-beta signaling
Source: PLoS One. 2025 Jul 10;20(7):e0327915. doi: 10.1371/journal.pone.0327915 (PMC12244532; doi:10.1371/journal.pone.0327915)
Supplement: S1 Table — (DOCX) [file pone.0327915.s009.docx]

**S1 Table. Sequences of RNA and DNA Oligonucleotides**

| **Name** | **Sense Strand/Sense Primer (5'-3')** | **Antisense Strand/Antisense Primer (5'-3')** |
| --- | --- | --- |
| **miRNA and siRNA duplexes** | | |
| miR-122 | UGGAGUGUGACAAUGGUGUUUG | AACACCAUUGUCACACUCAUUU |
| siTGFBR1 | GGGUCUGUGACUACAACAUUU | AUGUUGUAGUCACAGACCCAG |
| siITGAV | CAGGAUGGUUUCAAUGAUAUU | UAUCAUUGAA ACCAUCCUGGU |
| siRBM47 | UCCCCAAGAUGAAGAAGCGCGAGGA | UUUCCUCGCGCUUCUUCAUCUUGGG |
| NC | UUGUACUACACAAAAGUACUG | GUACUUUUGUGUAGUACAGUU |
| **Primers for RT-PCR** | | |
| ITGAV | TGAGGTCTTTGCACGGTTTG | GGATTTGAGATGGGACTGCG |
| ITGB1 | GGATTCTCCAGAAGGTGGTTTCG | TGCCACCAAGTTTCCCATCTCC |
| ITGB3 | CATGGATTCCAGCAATGTCCTCC | TTGAGGCAGGTGGCATTGAAGG |
| ITGB5 | GCCTTTCTGTGAGTGCGACAAC | CCGATGTAACCTGCATGGCACT |
| ITGB6 | TCTCCTGCGTGAGACACAAAGG | GAGCACTCCATCTTCAGAGACG |
| ITGB8 | CTGTTTGCAGTGGTCGAGGAGT | TGCCTGCTTCACACTCTCCATG |
| RBM47 | TGGAGGATACGCAGGCTACATACC | GTGGTCTGTCTTCGTGCTGGTC |
| TGFBR1 | TTGGCAAAGGTCGATTTGGA | AGAGCTGAGTCCAAGTACCA |
| N-cadherin | TGGACCATCACTCGGCTTA | ACACTGGCAAACCTTCACG |
| E-cadherin | GCCCCATCAGGCCTCCGTTT | ACCTTGCCTTCTTTGTCTTTGTTGGA |
| Vimentin | CCTGAACCTGAGGGAAACTAA | GCAGAAAGGCACTTGAAAGC |
| GAPDH | GAGTCAACGGATTTGGTCGT | GACAAGCTTCCCGTTCTCAG |
| **Primers for cloning** | | |
| RBM47-3'UTR | GATTTATTTCTGAATTATTTT | CAACCCTTAATGCCAGCAGA |
| ITGAV-PMT | CCCCGCTAGCGGAACTCCCGGTCTCAAGCA | CCCCCTCGAGCGGCCCGCCGCCTGCGCGCG |
| ITGAV-CDS | TCCGATTCCAAACTGGGAGC | TGTTCTTCTTGAGGTGGCCG |
| ITGAV-3’UTR | AGGCTTTACTGCTGATAGTGCT | GGTTGGCTTGAAGGATTTGACC |
| **Primers for RIP** | | |
| ITGAV-5’UTR | GAGCCGGAGGGAAGCAAAG | GCAGGCAAGAGGGCTGAG |
| ITGAV-CDS | GGAGCATCTGTGAGGTCGAA | AAAGCTACCAGGACCACCAAG |
| ITGAV-3’UTR | ACAGGAACCTGGACCCCTTA | TCTGACAGCCGAGACTGATT |
