# Supplementary material for: MiR-122 promotes metastasis of hepatoma cells by modulating RBM47-integrin alpha V-TGF-beta signaling
Source: PLoS One. 2025 Jul 10;20(7):e0327915. doi: 10.1371/journal.pone.0327915 (PMC12244532; doi:10.1371/journal.pone.0327915)
Supplement: S2 File — (PPTX) [file pone.0327915.s011.pptx]

## Slide 1
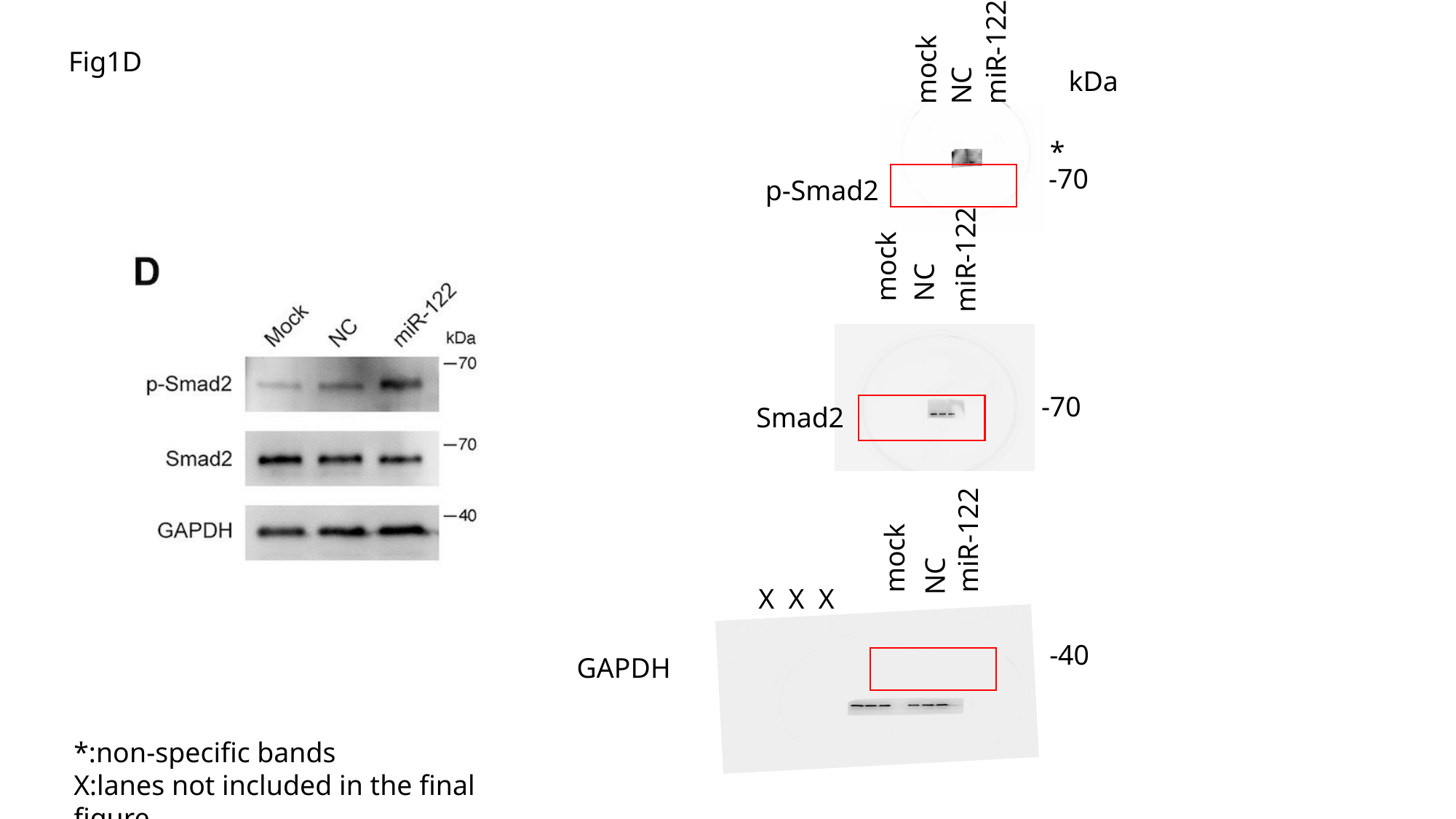

NC
miR-122
Fig1D
mock
kDa
*
-70
p-Smad2
miR-122
mock
NC
-70
Smad2
miR-122
mock
NC
X X X
-40
GAPDH
*:non-specific bands
X:lanes not included in the final figure

## Slide 2
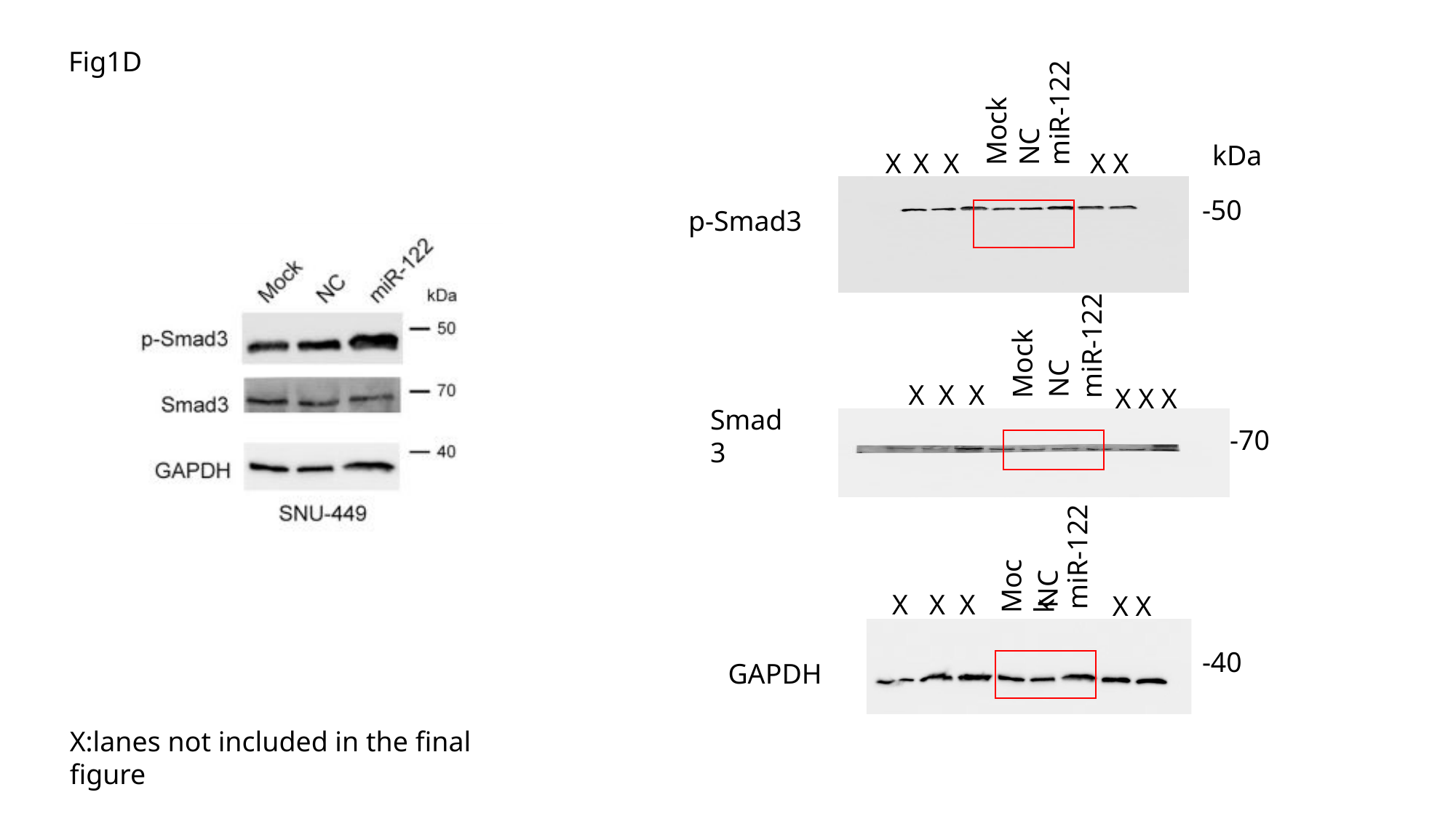

Mock
NC
miR-122
Fig1D
kDa
X
 X X
X X
-50
p-Smad3
miR-122
NC
Mock
X X X
X X X
Smad3
-70
miR-122
NC
Mock
X X X
X X
-40
GAPDH
X:lanes not included in the final figure

## Slide 3
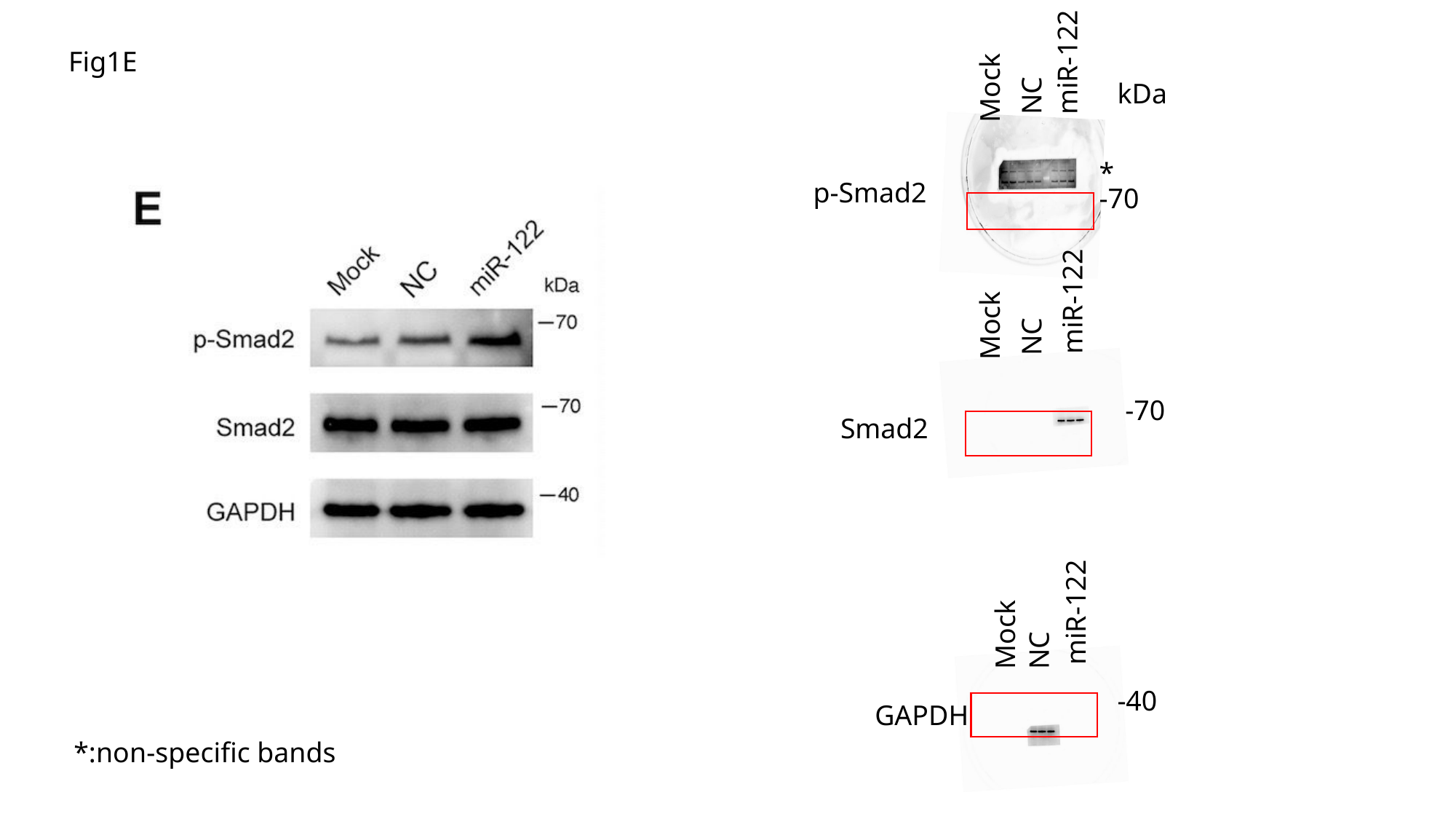

Mock
miR-122
Fig1E
NC
kDa
*
p-Smad2
-70
Mock
miR-122
NC
-70
Smad2
Mock
miR-122
NC
-40
GAPDH
*:non-specific bands

## Slide 4
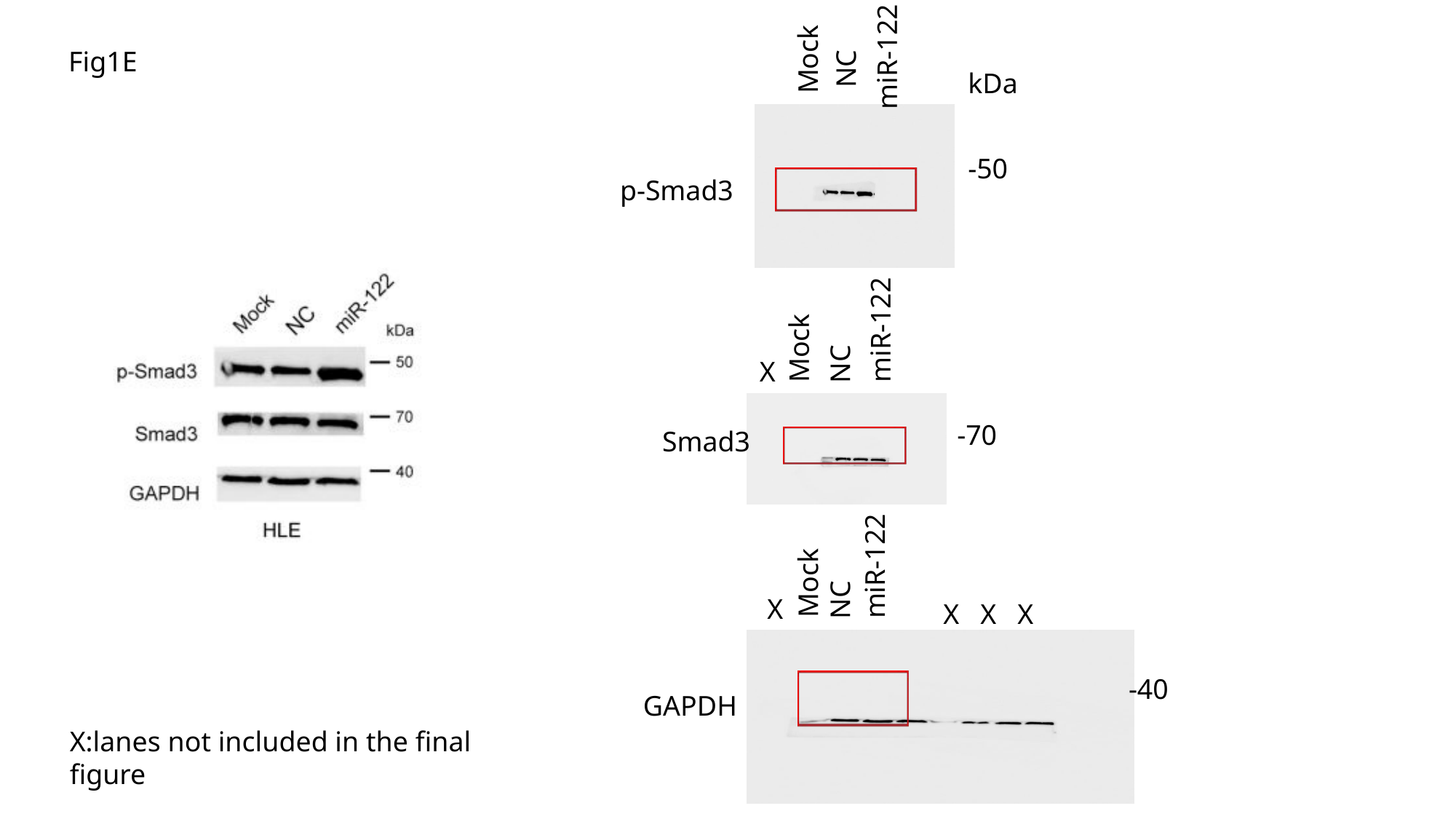

NC
miR-122
Mock
Fig1E
kDa
-50
p-Smad3
miR-122
Mock
NC
X
-70
Smad3
miR-122
Mock
NC
X
X X X
-40
GAPDH
X:lanes not included in the final figure

## Slide 5
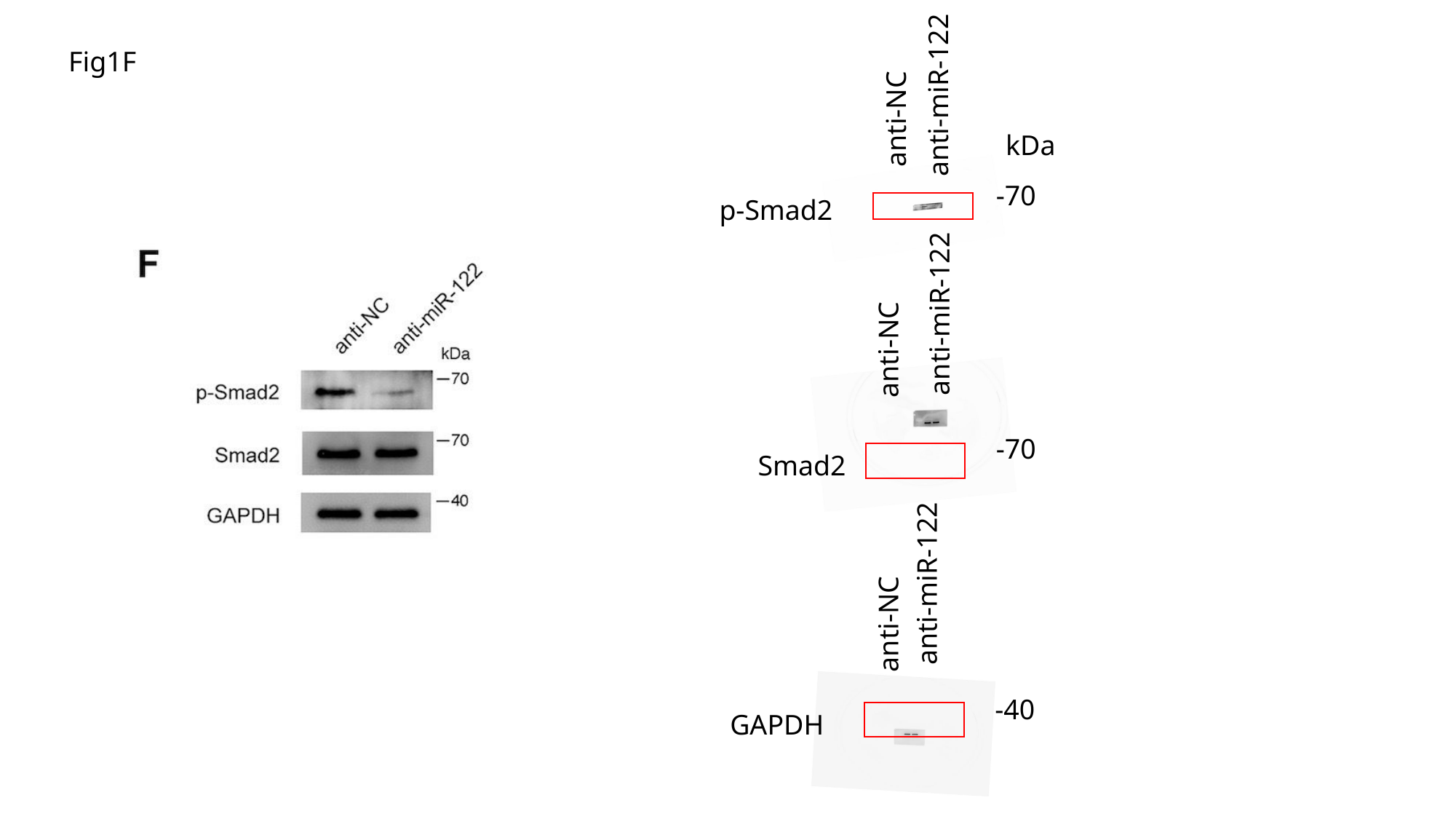

Fig1F
anti-miR-122
anti-NC
kDa
-70
p-Smad2
anti-miR-122
anti-NC
-70
Smad2
anti-miR-122
anti-NC
-40
GAPDH

## Slide 6
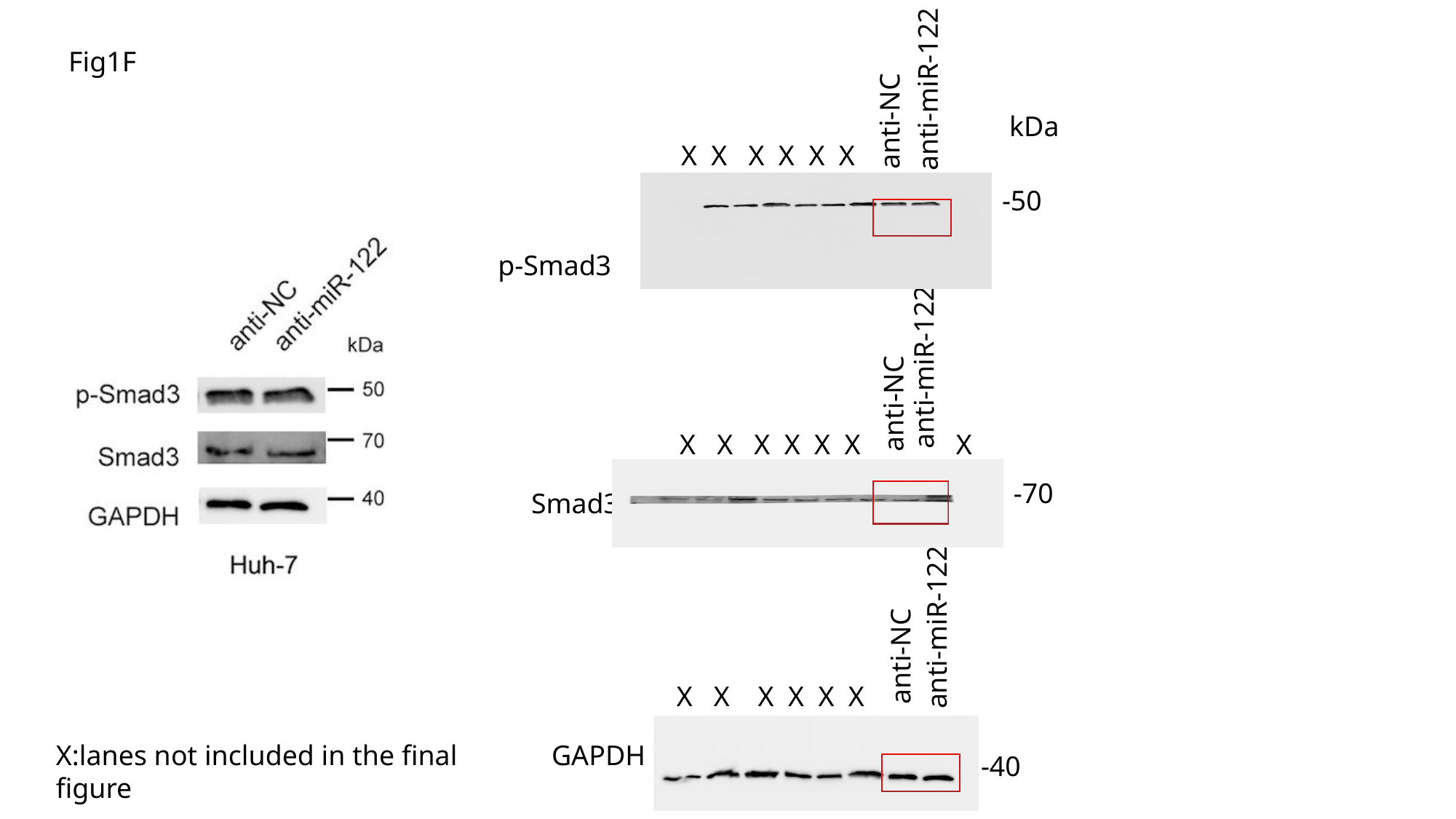

Fig1F
anti-miR-122
anti-NC
kDa
 X X X X X X
-50
-70
p-Smad3
anti-miR-122
anti-NC
X X X X X X
X
-70
Smad3
anti-miR-122
anti-NC
X X X X X X
X:lanes not included in the final figure
GAPDH
-40

## Slide 7
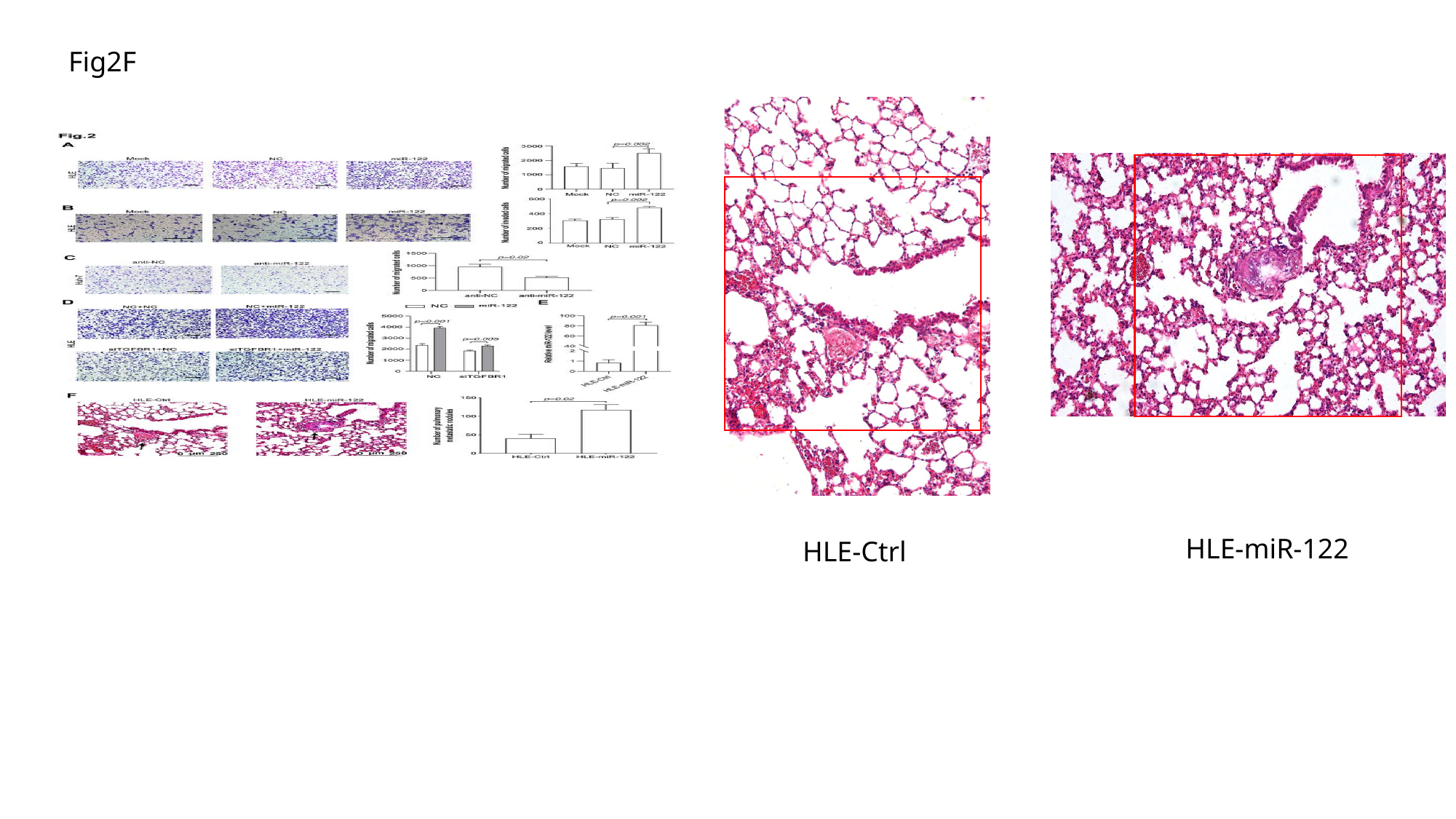

Fig2F
HLE-miR-122
HLE-Ctrl

## Slide 8
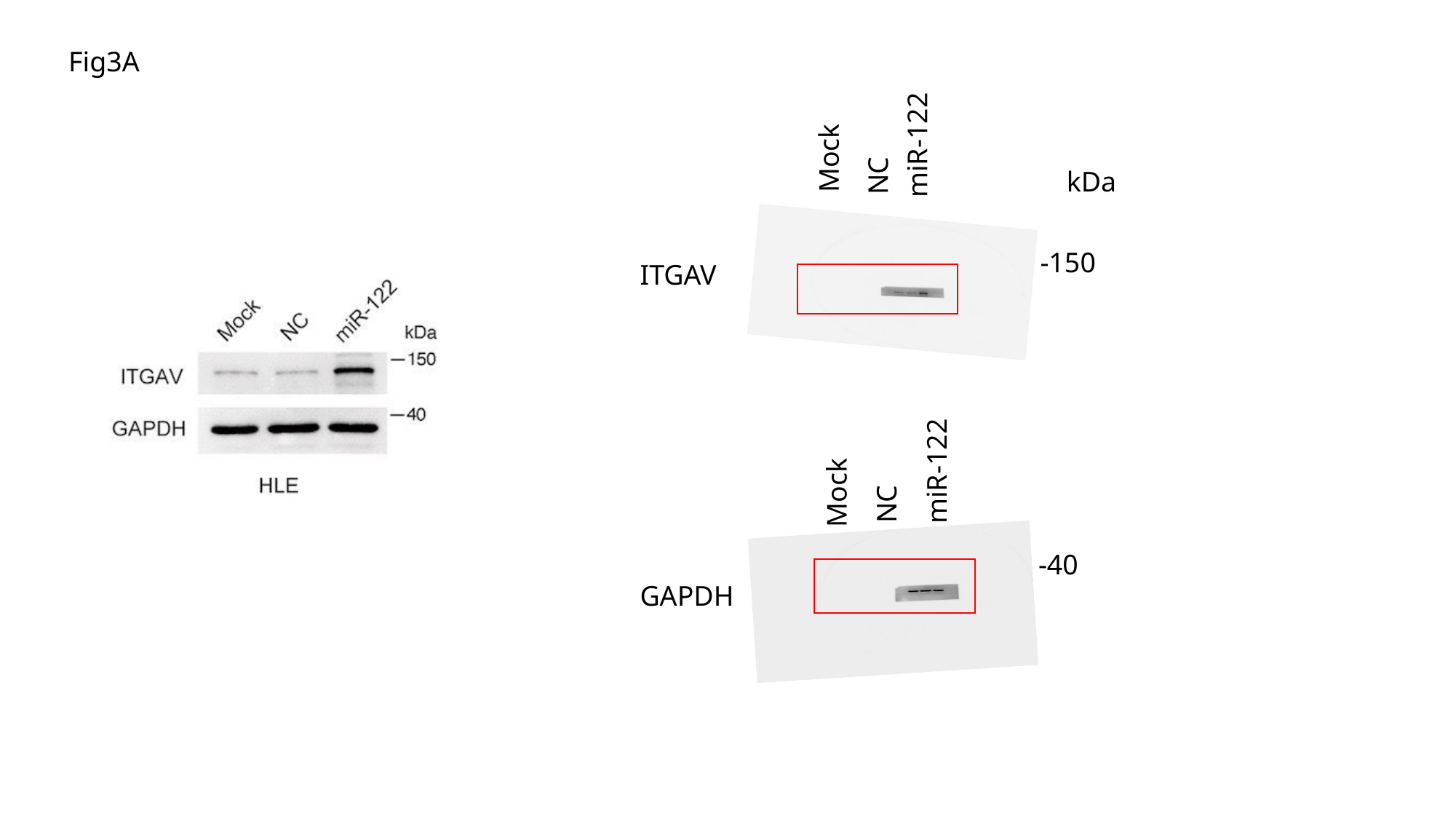

Fig3A
Mock
miR-122
NC
kDa
-150
ITGAV
Mock
miR-122
NC
-40
GAPDH

## Slide 9
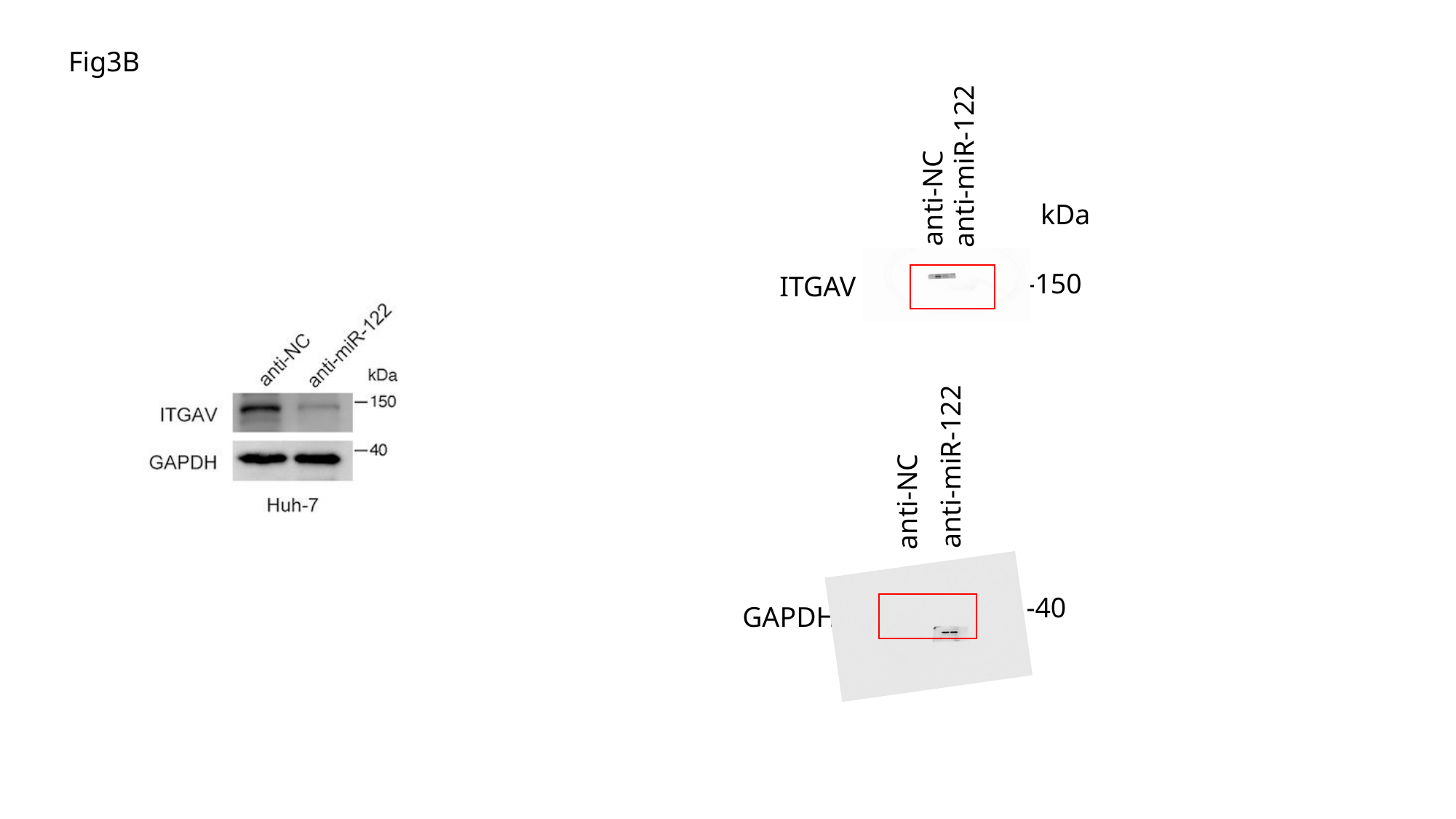

Fig3B
anti-miR-122
anti-NC
kDa
-150
ITGAV
anti-miR-122
anti-NC
-40
GAPDH

## Slide 10
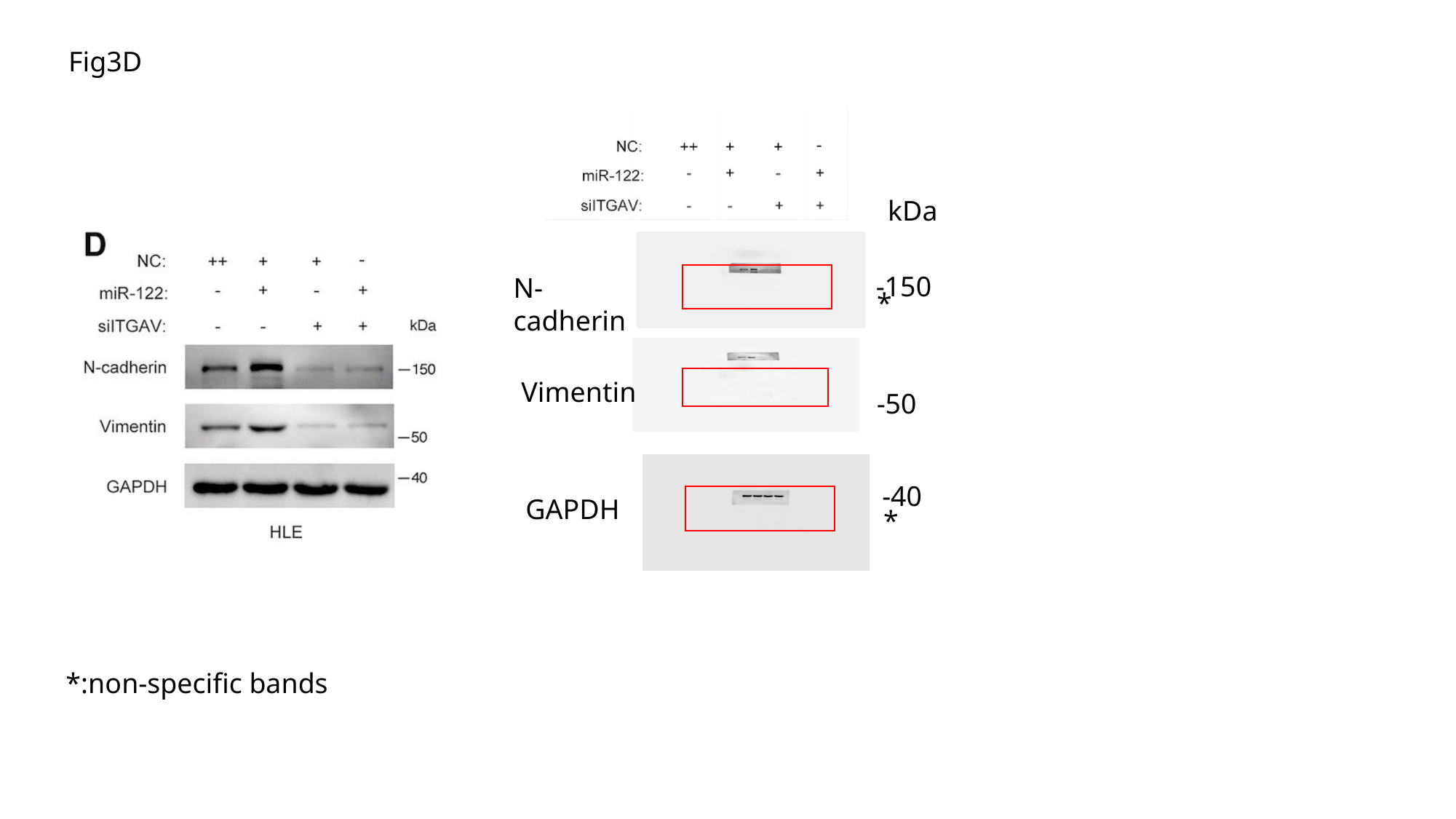

Fig3D
kDa
-150
N-cadherin
*
Vimentin
-50
-40
GAPDH
*
*:non-specific bands

## Slide 11
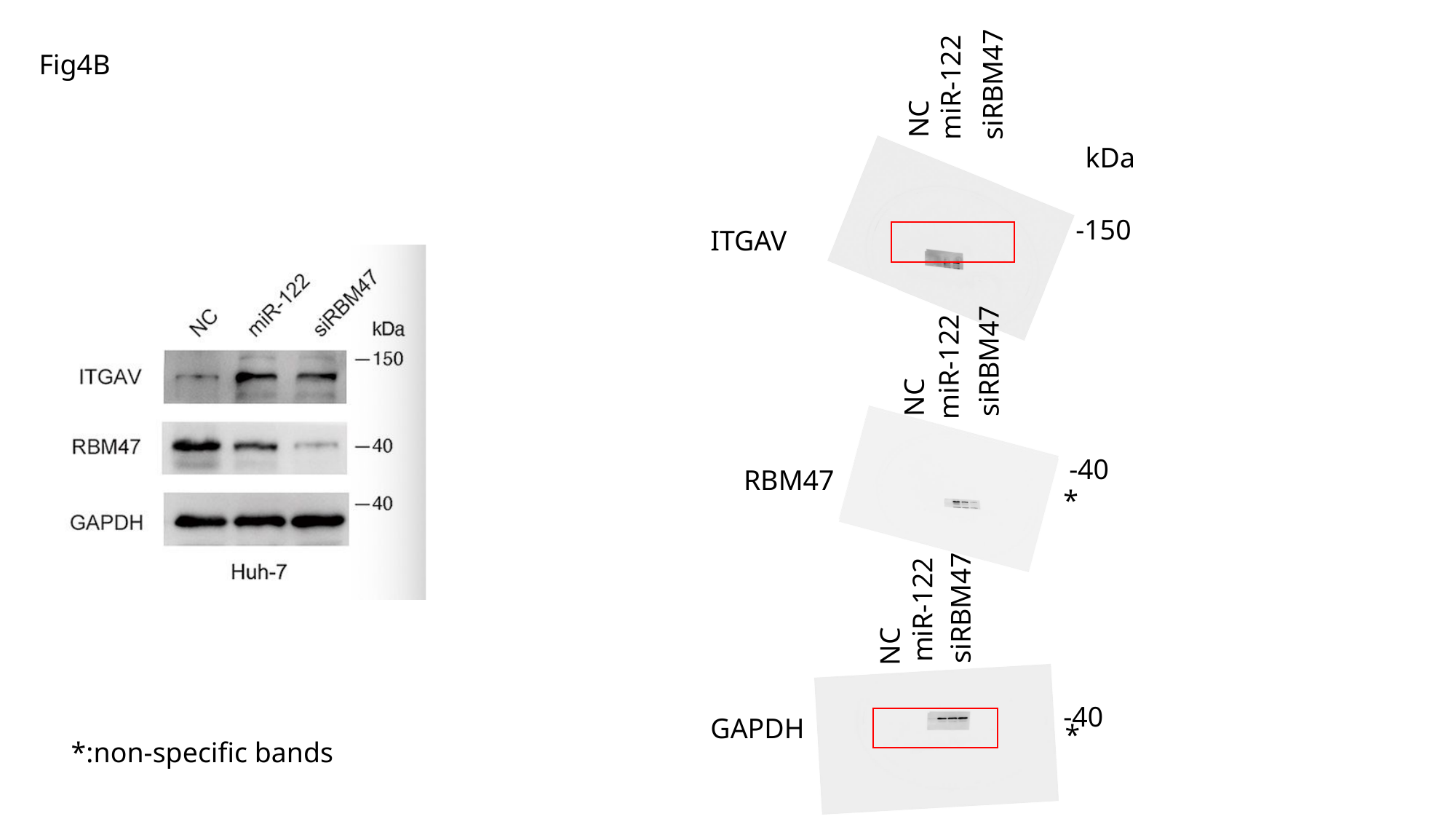

Fig4B
miR-122
NC
siRBM47
kDa
-150
ITGAV
miR-122
NC
siRBM47
-40
RBM47
*
miR-122
siRBM47
NC
-40
GAPDH
*
*:non-specific bands

## Slide 12
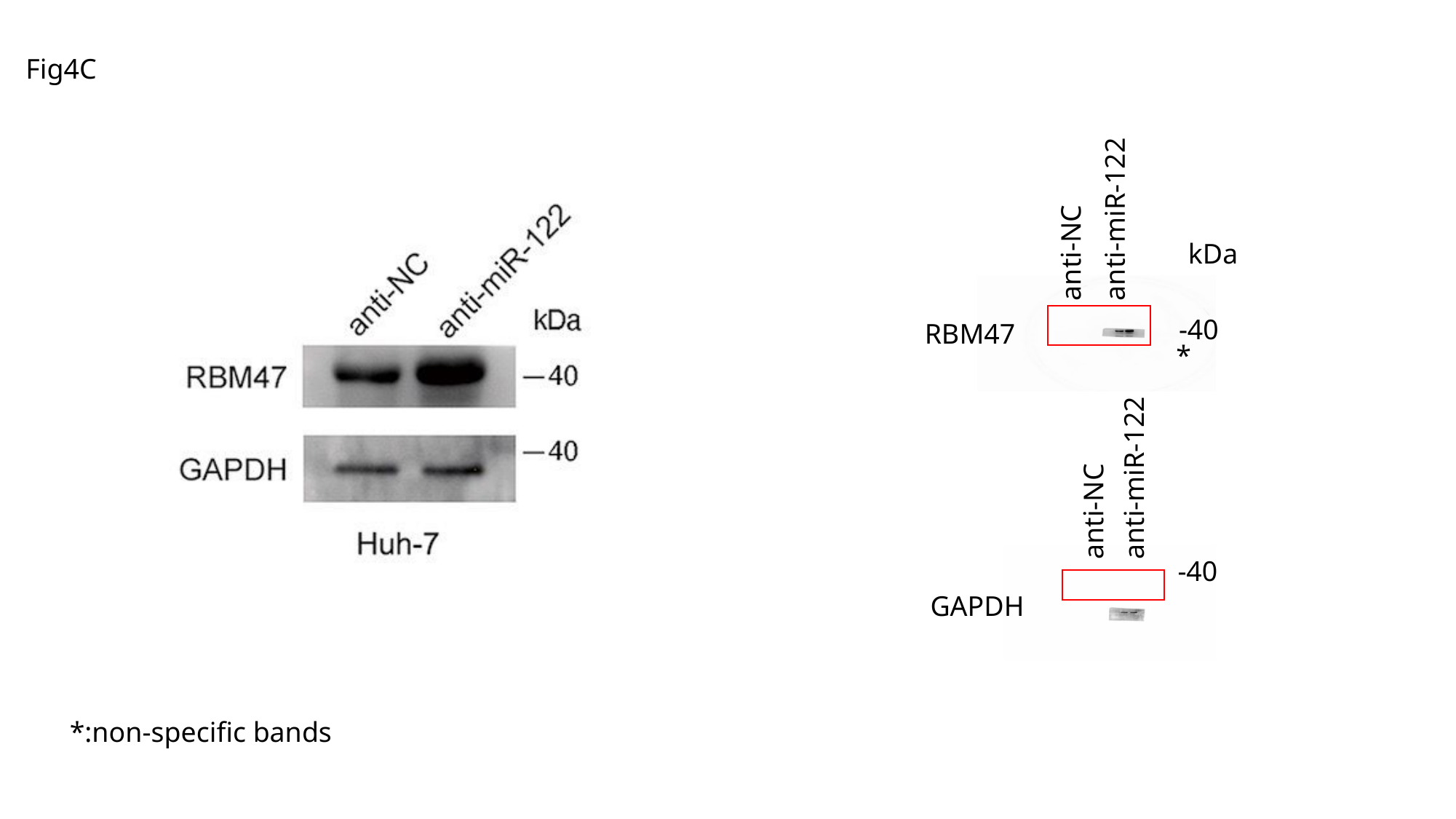

Fig4C
anti-miR-122
anti-NC
kDa
-40
RBM47
*
anti-miR-122
anti-NC
-40
GAPDH
*:non-specific bands

## Slide 13
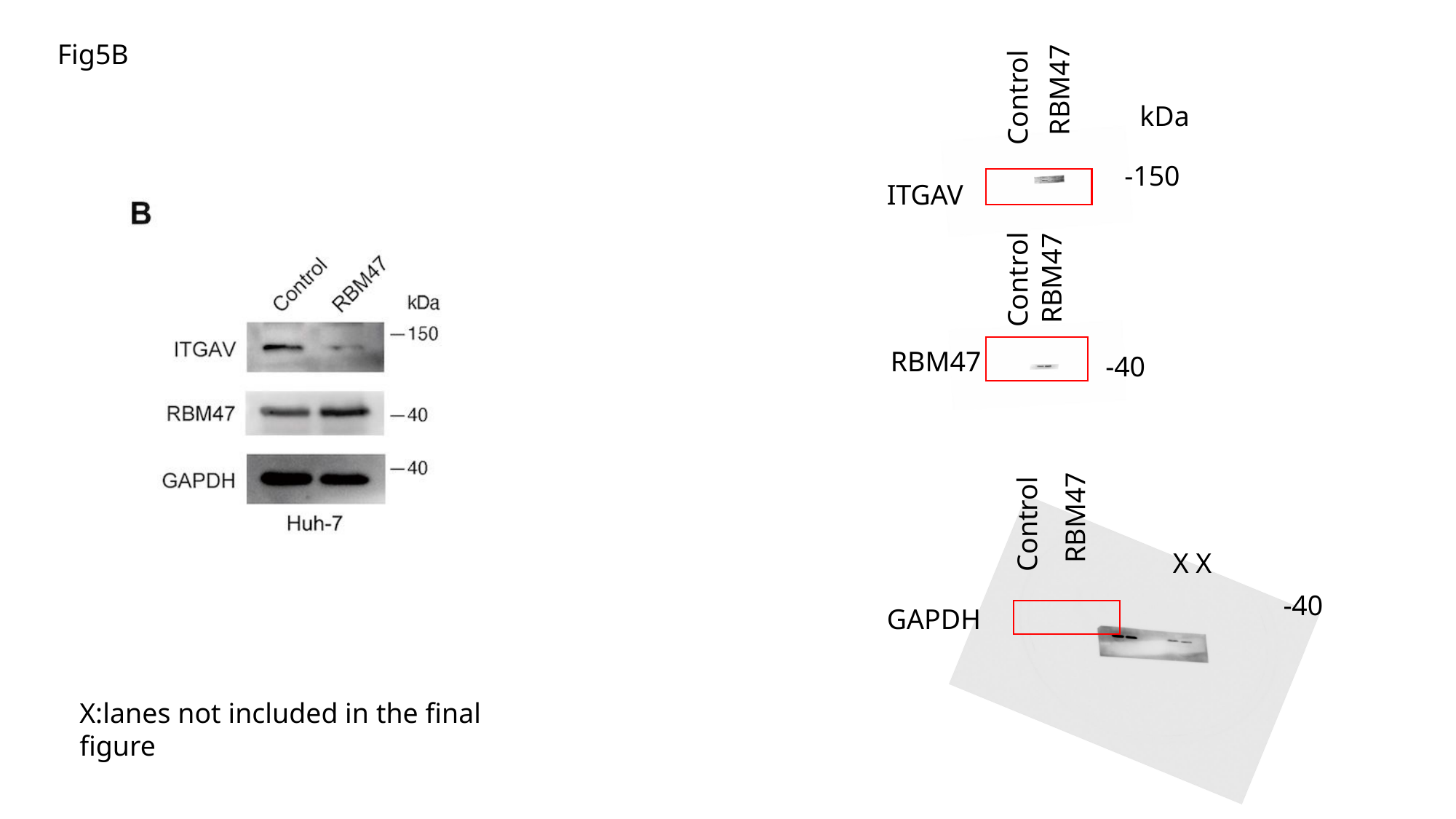

Fig5B
RBM47
Control
kDa
-150
ITGAV
RBM47
Control
RBM47
-40
RBM47
Control
X X
-40
GAPDH
X:lanes not included in the final figure

## Slide 14
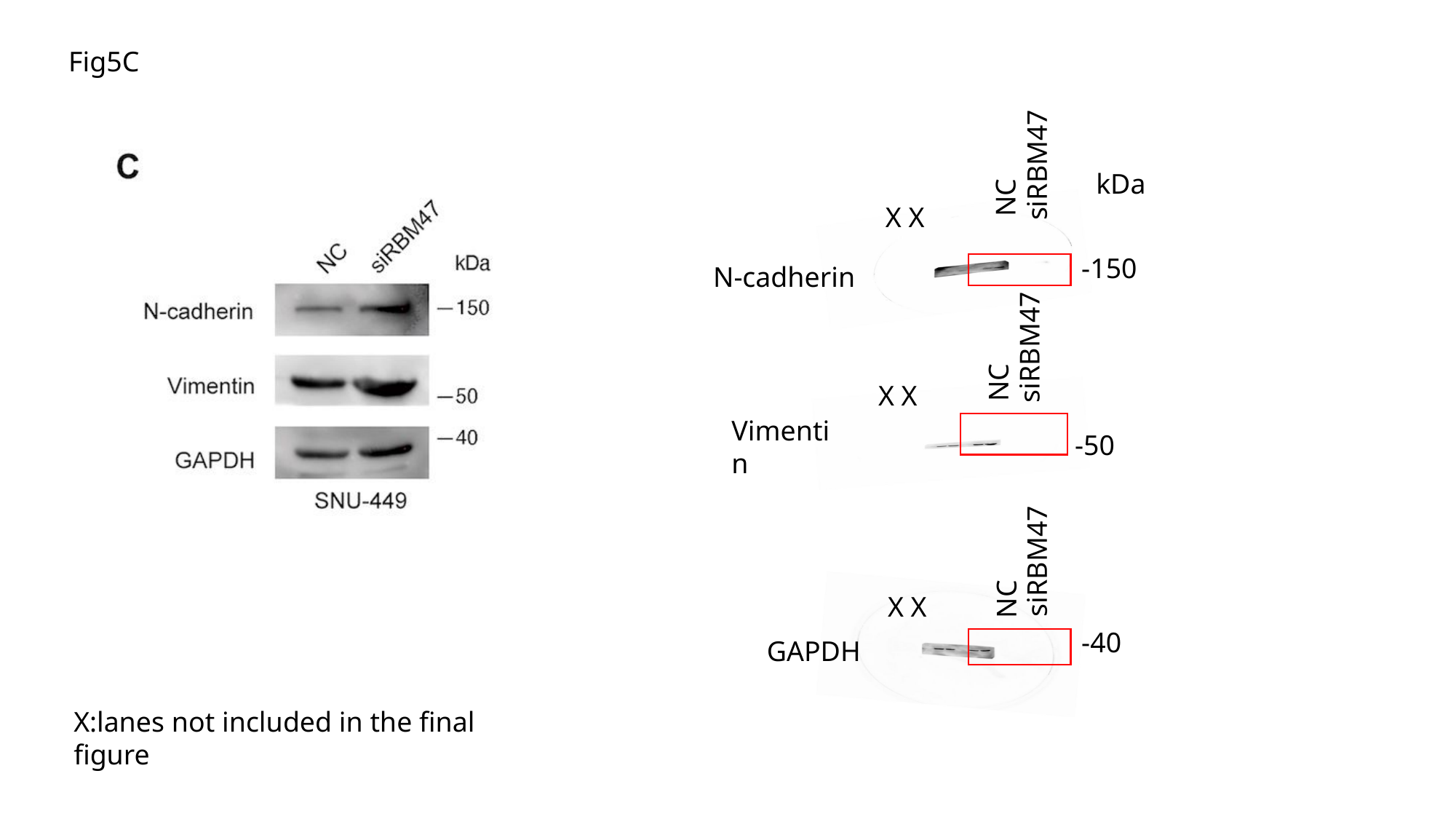

NC
siRBM47
Fig5C
siRBM47
kDa
 X X
-150
N-cadherin
NC
X X
Vimentin
-50
siRBM47
NC
X X
-40
GAPDH
X:lanes not included in the final figure

## Slide 15
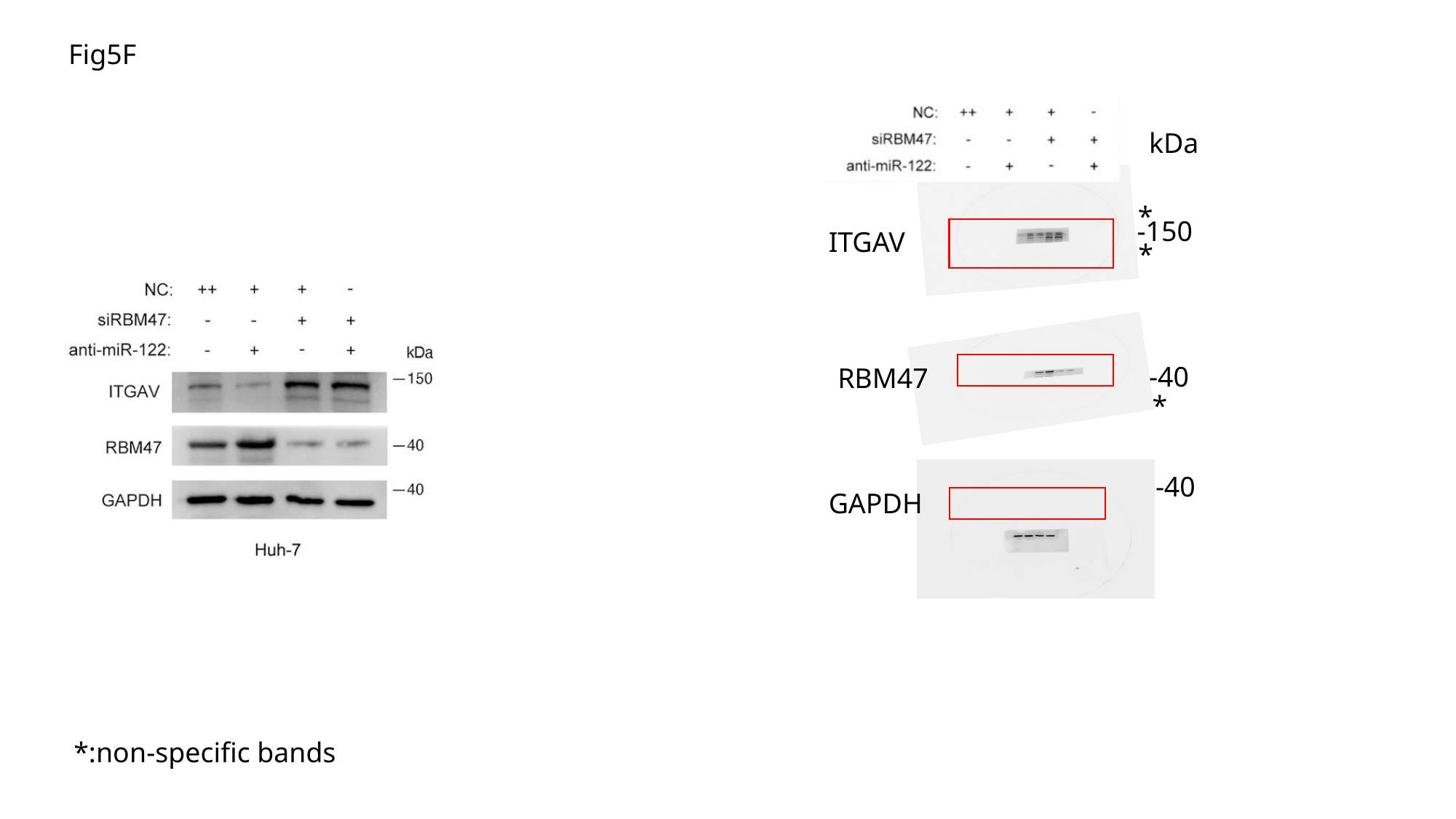

Fig5F
kDa
*
-150
ITGAV
*
-40
RBM47
*
-40
GAPDH
*:non-specific bands

## Slide 16
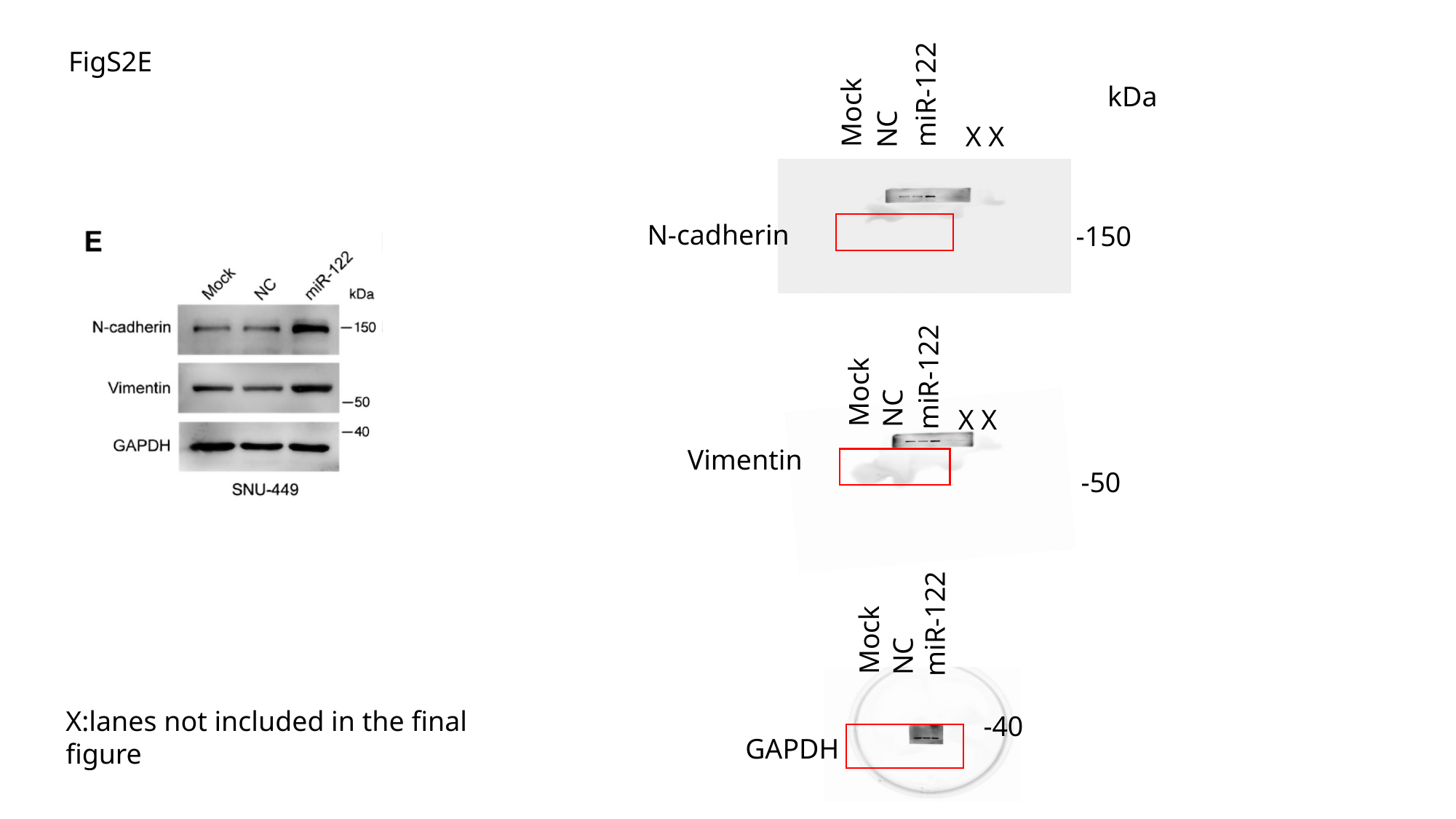

FigS2E
Mock
miR-122
kDa
NC
X X
N-cadherin
-150
Mock
miR-122
NC
X X
Vimentin
-50
Mock
miR-122
NC
X:lanes not included in the final figure
-40
GAPDH

## Slide 17
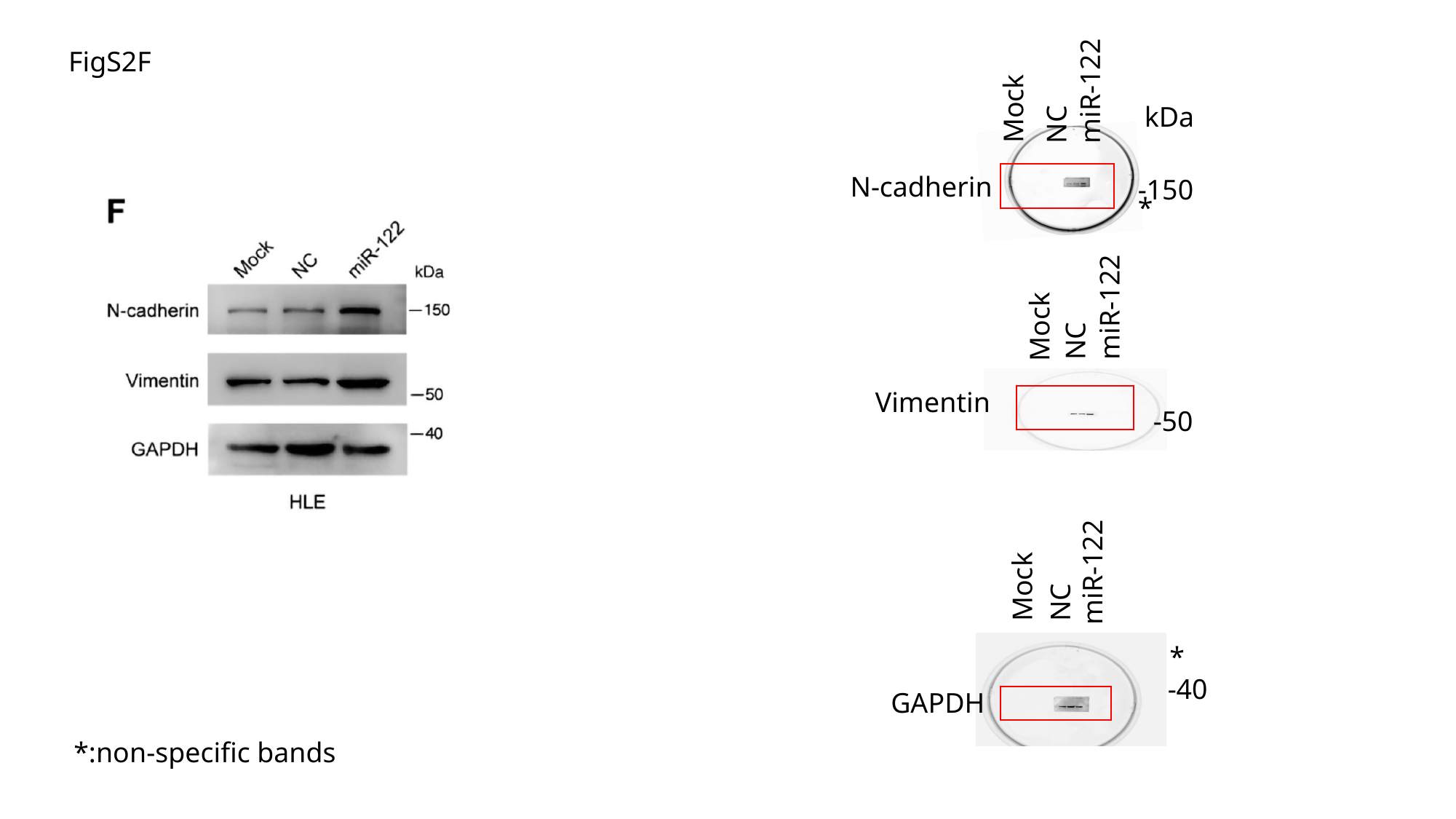

FigS2F
miR-122
Mock
NC
kDa
N-cadherin
-150
*
miR-122
Mock
NC
Vimentin
-50
miR-122
Mock
NC
*
-40
GAPDH
*:non-specific bands

## Slide 18
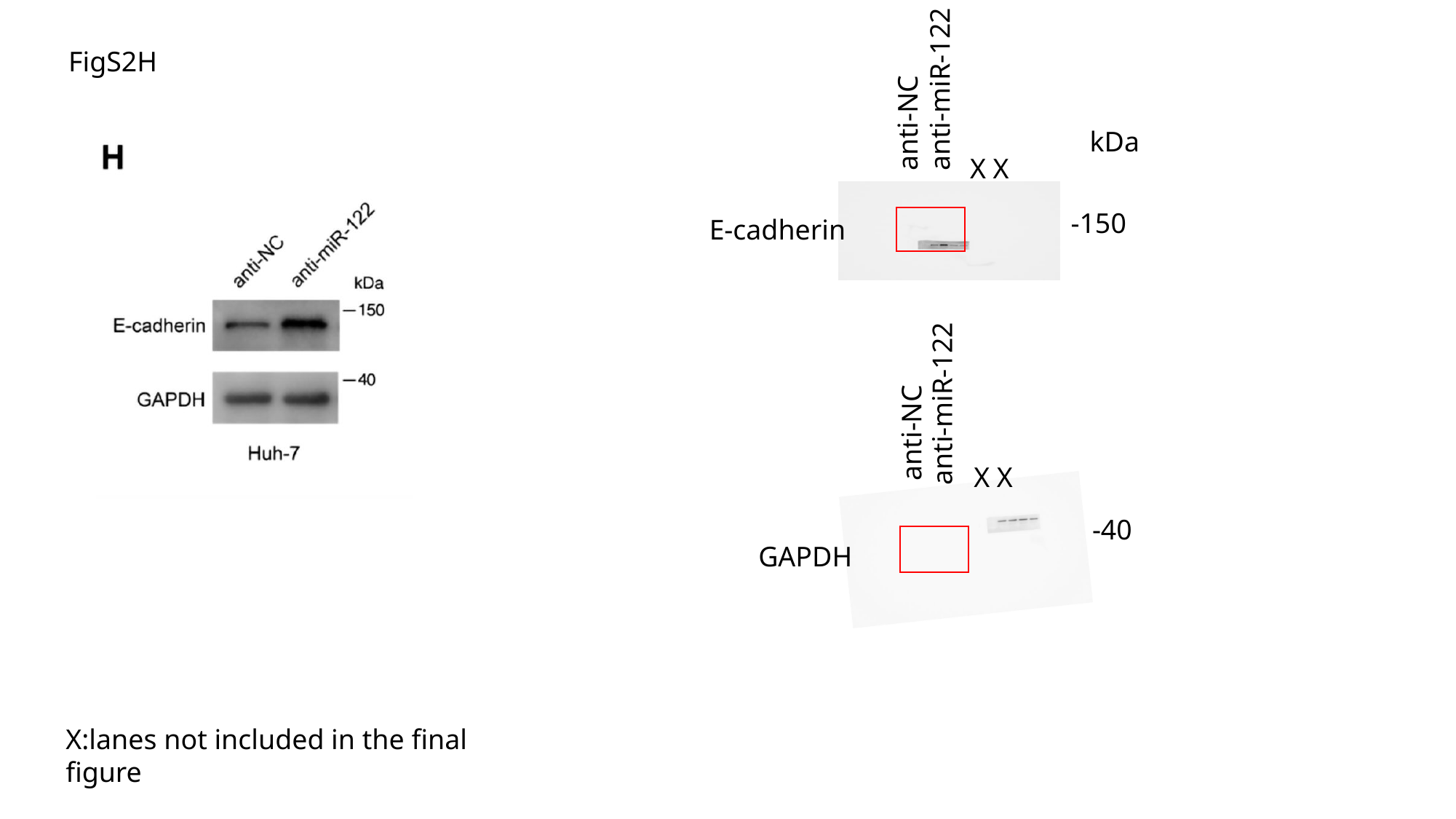

FigS2H
anti-miR-122
anti-NC
kDa
X X
-150
E-cadherin
anti-miR-122
anti-NC
X X
-40
GAPDH
X:lanes not included in the final figure

## Slide 19
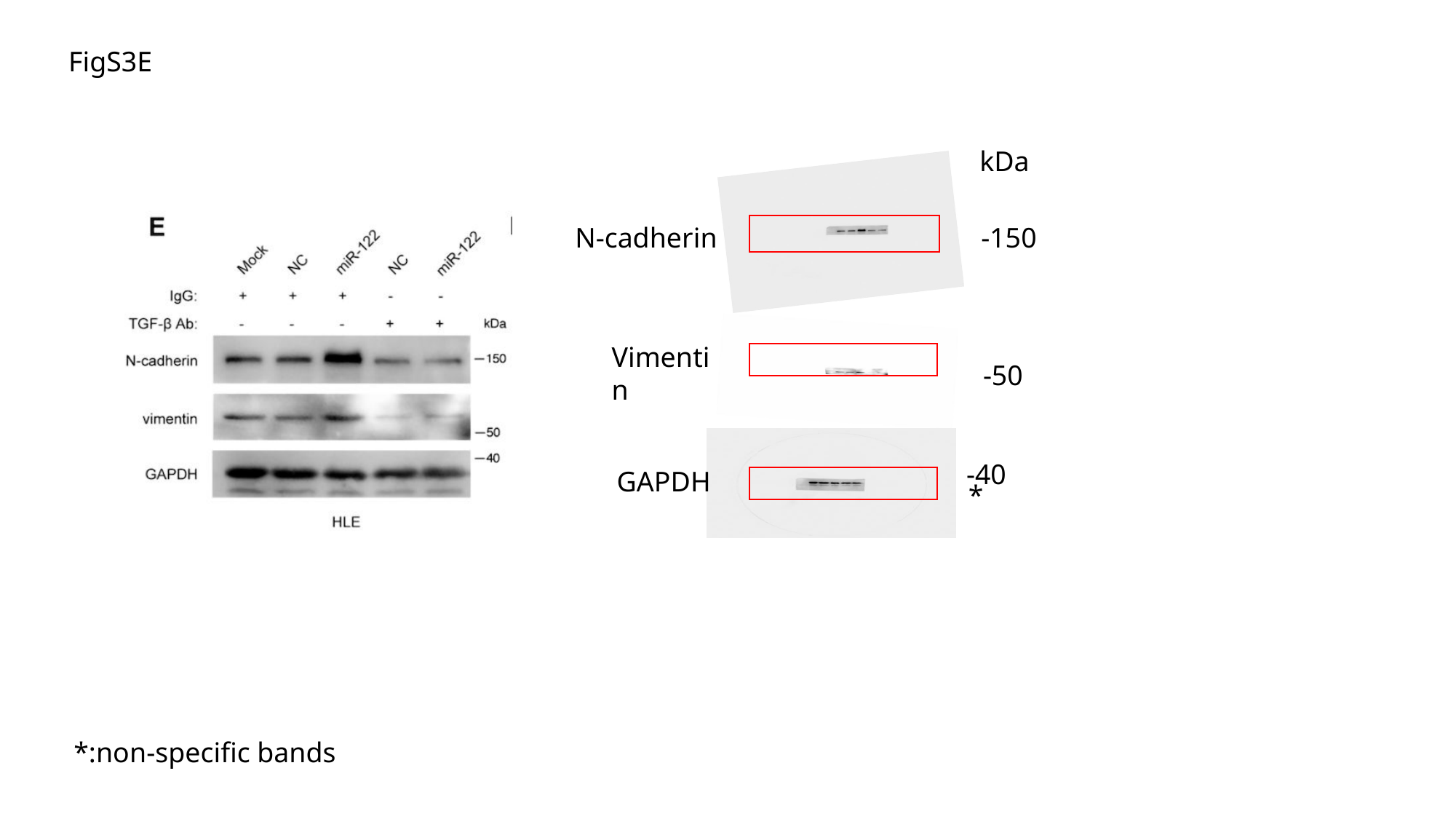

FigS3E
kDa
N-cadherin
-150
Vimentin
-50
-40
GAPDH
*
*:non-specific bands

## Slide 20
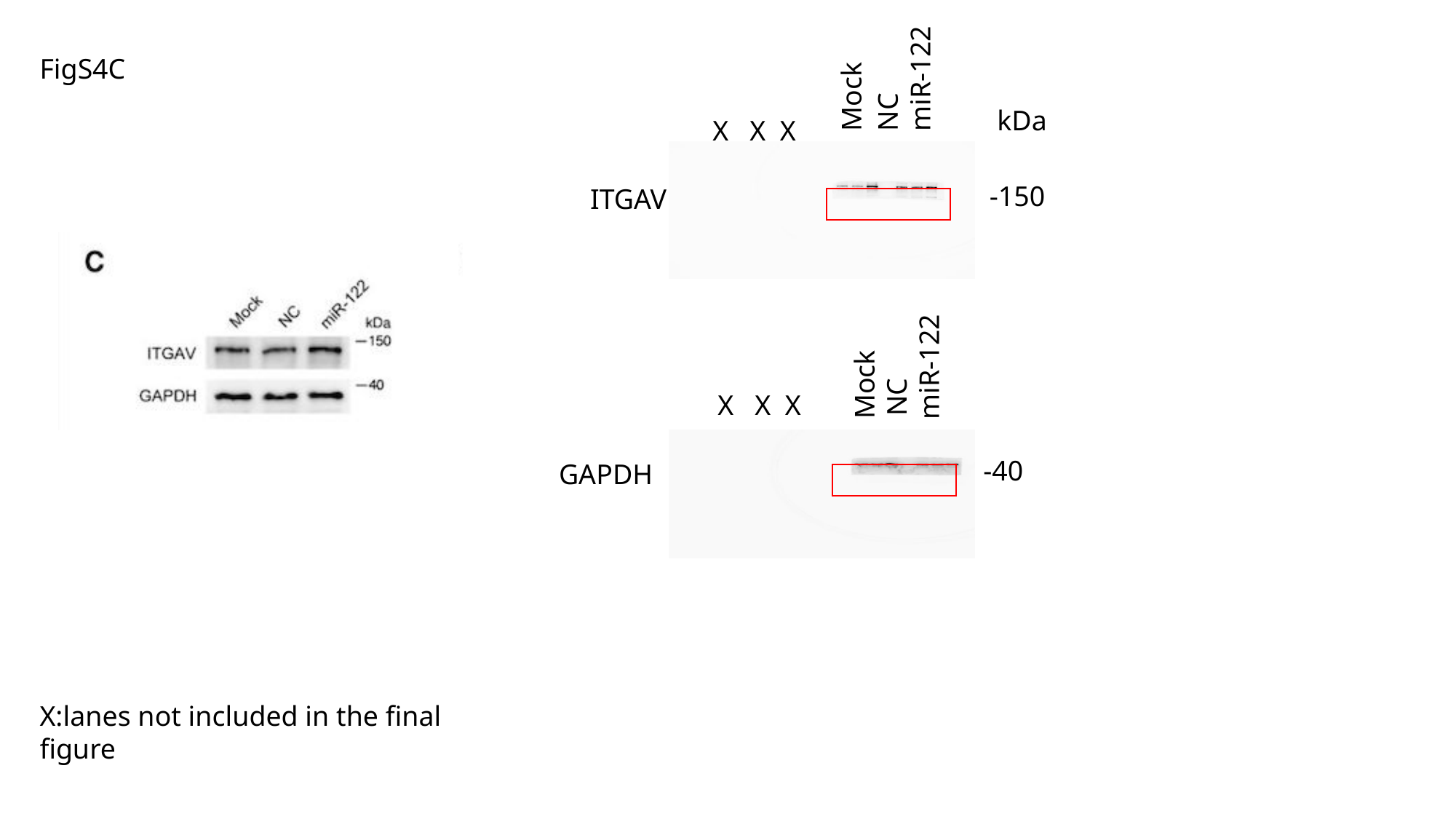

Mock
miR-122
FigS4C
NC
kDa
X X X
-150
ITGAV
Mock
miR-122
NC
X X X
-40
GAPDH
X:lanes not included in the final figure

## Slide 21
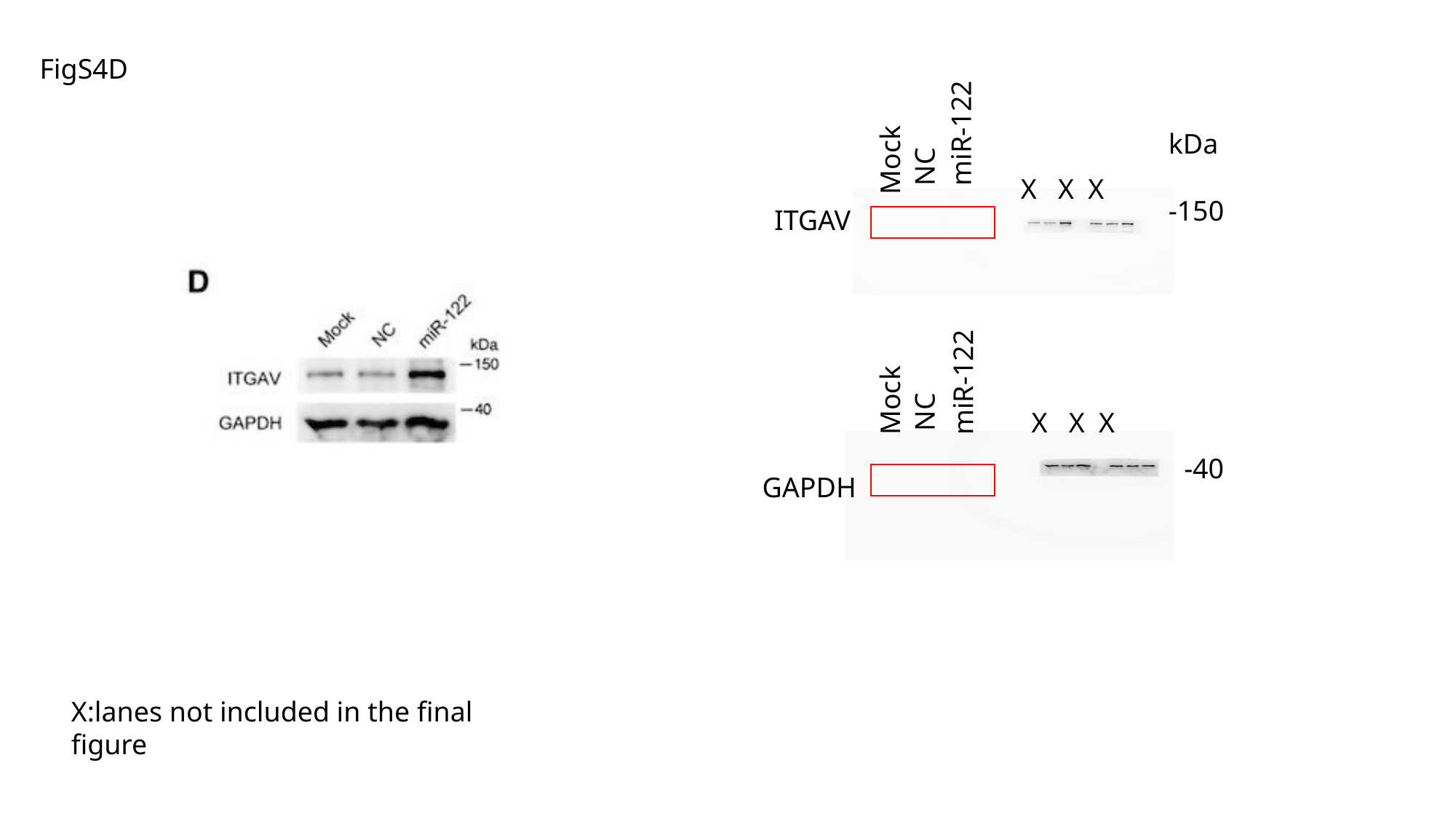

FigS4D
miR-122
NC
kDa
Mock
X X X
-150
ITGAV
miR-122
NC
Mock
X X X
-40
GAPDH
X:lanes not included in the final figure

## Slide 22
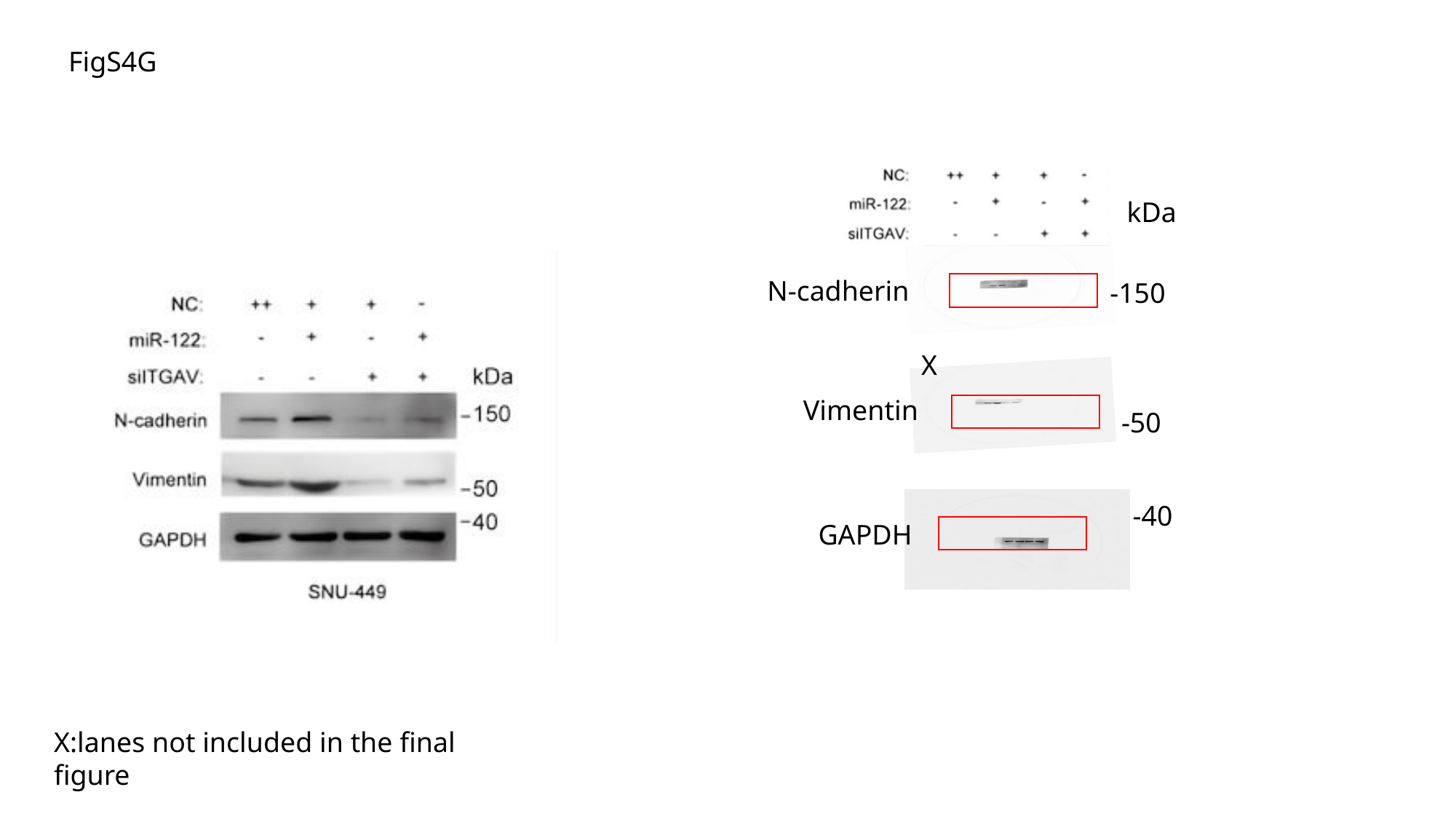

FigS4G
kDa
N-cadherin
-150
X
Vimentin
-50
-40
GAPDH
X:lanes not included in the final figure

## Slide 23
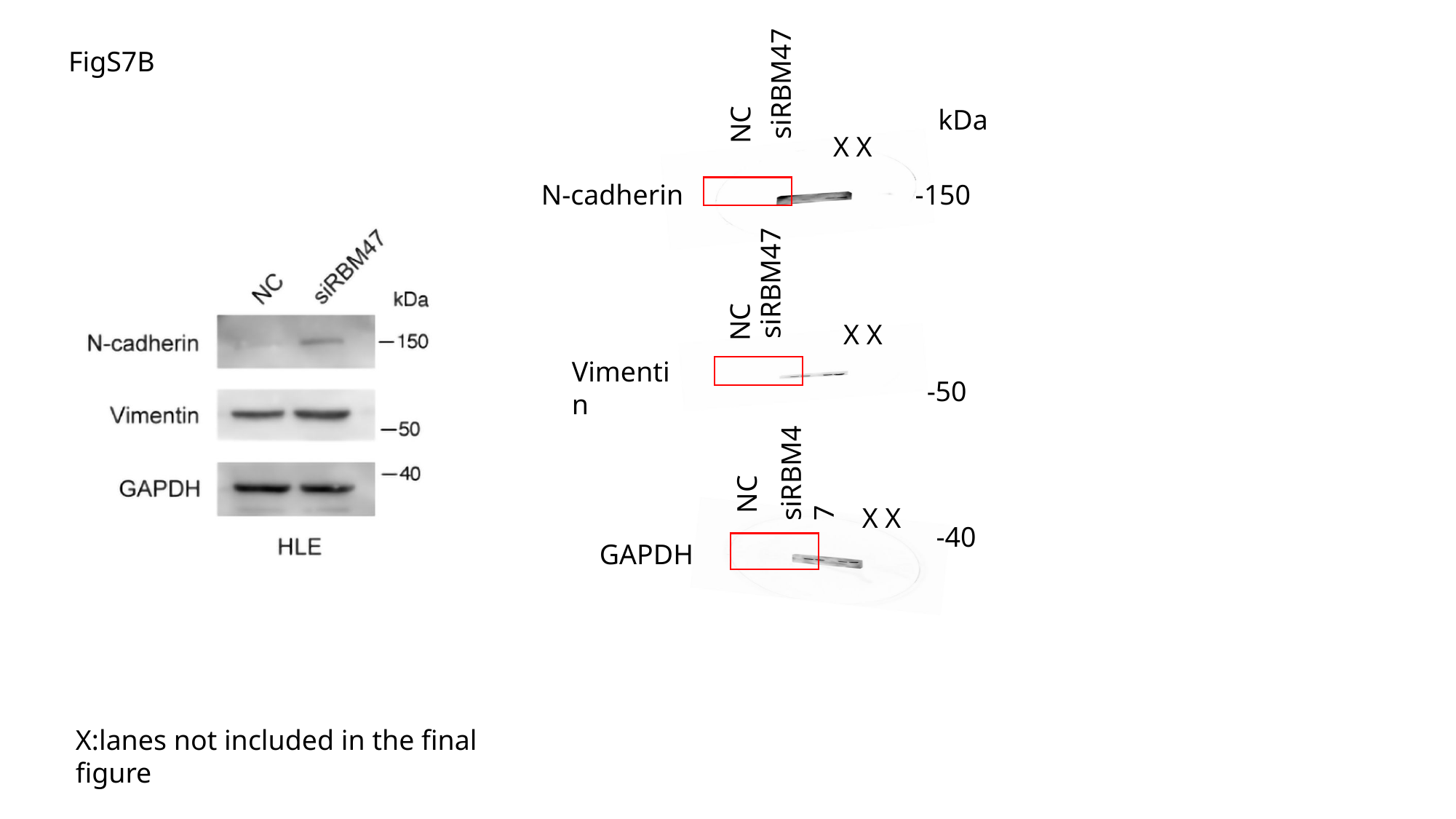

FigS7B
siRBM47
NC
kDa
X X
-150
N-cadherin
siRBM47
NC
X X
Vimentin
-50
siRBM47
NC
X X
-40
GAPDH
X:lanes not included in the final figure
