# Supplementary material for: MiR-122 promotes metastasis of hepatoma cells by modulating RBM47-integrin alpha V-TGF-beta signaling
Source: PLoS One. 2025 Jul 10;20(7):e0327915. doi: 10.1371/journal.pone.0327915 (PMC12244532; doi:10.1371/journal.pone.0327915)
Supplement: S3 File — (DOC) [file pone.0327915.s012.doc]

**Supplementary Materials and Methods**

**Reagents**

The reagents used were as follows: rabbit polyclonal antibodies against ITGAV (A2091, ABclonal, Wuhan, China, 1:1500 dilution), RBM47 (23902-1-AP, Proteintech, Wuhan, China, 1:1500 dilution); Smad2 (#5339, 1:1500 dilution), phospho-Smad2-Ser465/Ser467 (#18338, 1:1500 dilution), N-cadherin (#13116, 1:1500 dilution), E-cadherin (#3195, 1:1500 dilution), and vimentin (#5741, , 1:1500 dilution, Cell Signaling Technology, MA, USA); [phospho-Smad3-Ser213](http://www.abways.cn/showproduct.asp?cid=CY6484) (CY6484, abways, Shanghai, China, 1:1500 dilution); rabbit monoclonal antibody against Smad3 (CY5013, abways, 1:1500 dilution); mouse monoclonal antibodies against GAPDH (AC002, ABclonal, 1:1500 dilution); recombinant TGF-beta 1 Protein (10804-HNAC, Sino Biological, Beijing, China); Dactinomycin (Sigma-Aldrich, St. Louis, MO, USA); Lipofectamine RNAiMAX, and Lipofectamine 2000 (Invitrogen, Carlsbad, CA, USA); Dual-Luciferase Reporter Assay System (Promega, Madison, WI, USA); restriction enzymes (NEB, NE, USA); CCK-8 (C0037, Beyotime, Guangzhou, China); RNasin (10603), and SYBR Green qPCR kit (11196, Yeasen, Shanghai, China); Hairpin-it^TM^ miRNAs RT-PCR Quantitation Kit (GenePharma, Shanghai, China); Protein A/G Plus–agarose (Thermo Fisher, Carlsbad, CA, USA); DMEM and RPMI1640 (Hyclone, Logan, UT, USA); fetal bovine serum (Gibco, Grand Island, NY, USA).

**Cell lines**

HEK293T, Huh-7, HLE and SK-Hep-1 cell lines were cultured in DMEM containing 10% FBS; SNU-449 and CAF cell line were maintained in RPMI with 10% FBS. All cells were maintained in the conditions of 37℃, 5% CO_2_ and saturated humidity.

**Plasmids**

The details of plasmid construction are as follows. To assess the TGF-β pathway activity, the Smad binding element (SBE) sequences were inserted into pGL3-Basic to construct p-SBE. To verify miR-122-targeted 3’UTR, a p-RBM47-3’UTR-WT was constructed by inserting a 446-bp 3’UTR fragment of human RBM47, which contains putative binding sites of miR-122, into the *Eco*RI and *Xba*I sites downstream of the stop codon of firefly luciferase in pGL3cm, which was previously produced based on pGL3-control (Promega). The p-RBM47-3’UTR-MUT plasmid, which carried the mutated sequence in the complementary site for the seed region of miR-122, was created by fusion PCR based on p-RBM47-3’UTR-WT vector. To dissect the regulatory effect of miR-122 on ITGAV, the genomic sequence of ITGAV promoter region (-1295/+207) was cloned into the *Nhe*Ⅰ and *Xho*Ⅰ sites upstream of the firefly luciferase in pGL3-Basic vector (Promega) to generate p-ITGAV-PMT. In research for the interaction between RBM47 and ITGAV mRNA, the vectors of p-CDS, p-3’UTR, p-3’UTR-dARE were constructed. Briefly, a 1000-bp CDS fragment upstream of and a 1000-bp 3’UTR fragment downstream of the stop codon of human ITGAV mRNA, and the same 3’UTR fragment without AU-rich elements (ARE), were cloned and inserted into the *Eco*RI and *Xba*I sites of pGL3cm, respectively, as the 3’UTR of the luciferase reporter.

**Luciferase reporter assay**

Both gain- and loss-of-function studies were performed to detect the effect of miR-122 on TGF-β pathway activity. For the gain-of-funtion analysis, HCC cell lines cultured in a 48-well plate were co-transfected with 10 ng of pRL-TK (Promega), 300 ng of p-SBE and 30 nM of either NC or miR-122 mimics. For the loss-of-function study, Huh-7 cells cultured in a 48-well plate were co-transfected with 5 ng of pRL-TK, 150 ng of p-SBE and 200 nM of either anti-NC or anti-miR-122. For the assay to verify miR-122-targeted 3’UTR, HEK293T cells grown in a 48-well plate were co-transfected with 10 ng of either p-RBM47-3’UTR-WT or MUT reporter, 2 ng of pRL-TK and 50 nM of either NC or miR-122 duplex. For the assay of ITGAV promoter activity, Huh-7 cells were co-transfected with 10 ng of pRL-TK, 300 ng of p-ITGAV-PMT and 30 nM miR-122/NC duplex, or 5 ng of pRL-TK, 150 ng of p-ITGAV-PMT and 150 ng of RBM47-expression/Ctrl plasmid. Both gain- and loss-of-function studies were used to dissect the interaction between RBM47 and ITGAV 3’UTR. For the gain-of-funtion analysis, Huh-7 cells were co-transfected with 5 ng of pRL-TK, 150 ng of p-CDS/3’UTR/3’UTR-dARE and 150 ng of RBM47-expression/Ctrl plasmids. For the loss-of-function study, Huh-7 cells were co-transfected with 10 ng of pRL-TK, 300 ng of p-CDS/3’UTR/3’UTR-dARE and 30 nM siRBM47/NC duplex. Cells were harvested 48h after cell transfection and analyzed using the Dual-Luciferase Reporter Assay System (Promega). Luciferase activity was detected by M200 microplate fluorescence reader (Tecan). *Renilla* luciferase was detected to correct the differences in both transfection and harvest efficiencies. Transfections were done in duplicates and repeated at least thrice in independent experiments.

**Analysis of gene expression**

Real-time quantitative RT-PCR (qPCR) were used to detect RNA levels. Total RNA from cells was extracted using the FastPure Cell/Tissue Total RNA Isolation Kit V2 (RC112-01, Vazyme) and reverse transcribed using Hifair® Ⅲ 1st Strand cDNA Synthesis SuperMix for qPCR (gDNA digester plus) (11141ES60, Yeasen ) following the manufactory’s instruction.

The expression level of miR-122 was quantified by a Hairpin-it^TM^ miRNAs RT-PCR Quantitation Kit (GenePharma) using U6 as a reference gene. The qPCR analyses for the mRNA levels of ITGAV, RBM47, TGFBR1, N-cadherin, E-cadherin and vimentin were performed by Hieff UNICON® qPCR SYBR Green Master Mix (Yeasen), using GAPDH as a reference gene. The temperature cycle profile for the qPCR reactions was 95 °C for 30 s and 40 cycles of 95 °C for 10 s and 60 °C for 30 s. The melting curve analysis was performed to verify the specificity of the PCR product immediately after amplification, as follows: heating to 95°C for 20 s, cooling to 60 °C for 20 s, followed by a temperature increase to 95°C with a transition rate of 0.11°C/s and the continuous detection of fluorescence. All qPCR reactions were performed on ABI7500 (Applied Biosystemd, USA), run in triplicate and repeated in three independent experiments. The cycle threshold (Ct) values did not differ more than 0.5 among the triplicate runs. Relative gene expression of target was normalized to the reference gene and calculated using 2^-ΔΔCt^ method. Sequences of primers are listed in S1 Table.

**Immunoblotting**

Cells were lysed using the lysis buffer (P0013C, Beyotime, China) containing protease and phosphatase inhibitors (P1005, Beyotime, China). The cell lysate were subjected to electrophoresis at constant pressure (80V for 30 minutes and 120V for 1 h) and then transferred to the PVDF membrane. Next, the membrane was blocked with 5% skimmed milk for 1 h and then incubated with antibodies diluted at 1:1500 overnight at 4℃. Subsequently, the membrane was washed with TBST solution three times for 10 minutes each time, and then incubated with the secondary antibodies goat anti-rabbit IgG (31460, ThermoFisher) or goat anti-mouse IgG (31430, ThermoFisher) at the dilution ratio of 1:5000 for 1 h at room temperature. The membrane was additionally washed with TBST solution three times for 10 min each time and developed using the ECL luminescent solution (P0018S, Beyotime, China).

***In vitro* assays of cell migration and invasion**

The migration and invasion of tumor cells were analyzed in 24-well Boyden chamber with 8 μm pore size polycarbonate membrane (Corning Glass Works, Corning, NY, USA). For invasion assay, the membrane was coated with 15 μg Matrigel to form a matrix barrier. All cell lines were reverse transfected with RNA duplex or RNA inhibitors. The cells (3.5x10^4^ for HLE and SK-Hep-1, and 4x10^4^ for SNU-449 and Huh-7) were suspended in 100 μl serum-free medium at 48 h post-transfection and added to the upper compartments of the chambers. The lower compartments were filled with 600 μl of medium with 10%FBS. After an incubation at 37 ℃ for 12 h (HLE and SK-Hep-1) or 24 h (SNU-449 and Huh-7), the cells remaining on the upper surfaces of the membrane were removed. The cells on the lower surfaces of the membrane were fixed by methanol, stained with crystal violet and counted. The statistical analyses were performed by counting cell numbers in four random fields per well, under a light microscope at a magnification of 150×. Representative images and the number of migrated cells per well were shown.

**Analysis of cell viability**

HLE and SNU-449 cells were transfected in 96-well plate with 10,000 cells per well and cultured for 48 hours. 100μl medium containing 10% CCK-8 were added to each well for 1 hour prior to submitting to assay. OD values were measured following the CCK-8 kit instruction.

**Hematoxylin-Eosin (H&E) staining**

The tissue sections were placed in an exclusive sectioning frame and immersed in 100% xylene (A530011, Sangon Biotech, China) for 10 min three times, followed by reacting with gradient ethanol (from 100% to 75%) for 3 min each. Subsequently, the sections were rinsed in distilled water in a glass beaker and maintained for approximately 2 min, and placed in hematoxylin staining solution in the assay kit (G1120, Solarbio, China) for about 5 min. Then the sections were immediately washed with tap water to remove excessive dyeing solution to develop the blue color, and placed in hydrochloric acid differentiation solution for 15 seconds, followed by rinsing in tap water for 5 mins. The specimens were stained by the eosin staining solution for 2 min, then rinsed in distilled water for 5 mins, and then dehydrated in gradient ethanol (from 75%-100%). After transparentized by 100% xylene, the sections were dried, sealed with neutral resin (G8590, Solarbio, China) and observed under an orthostatic microscope (Leica, Germany).

**RNA immunoprecipitation assays**

Huh-7 cells were cross-linked with 0.5% formaldehyde for 10 min at room temperature and glycine solution was added to quench the crosslink. Cells were washed with ice-cold 1×PBS, scraped in 1×PBS, and collected by centrifugation. Cell pellets were suspended in lysis buffer [25 mM Tris-HCl (pH 7.4), 150 mM NaCl, 1 mM EDTA, 1% NP-40 and 5% glycerol] with protease inhibitors and RNasin (1000 U/ml). The lysates were incubated with 3 μg of anti-RBM47 Antibody or its IgG isotype at 4℃ with rotation overnight. The RNA-protein complexes were precipitated and collected by Protein A/G Plus–agarose, and then digested using proteinase K for 1 h at 55 ℃. The purified RNAs were subsequently analyzed by RT-qPCR. The primers used were listed in S1 Table.
